# Mitochondrial reactive oxygen species promote cancer metastasis and tumor microenvironment immunosuppression through gasdermin D

**DOI:** 10.1038/s41420-025-02516-7

**Published:** 2025-05-06

**Authors:** Naijun Miao, Zhengchun Kang, Zhuning Wang, Wenyan Yu, Ting Liu, Ling-zhijie Kong, Ying Zheng, Changli Ding, Zhiyong Zhang, Chen Zhong, Qingliang Fang, Kaichun Li

**Affiliations:** 1 Department of Oncology, Shanghai Fourth People’s Hospital, Tongji University School of Medicine, Shanghai, 200434 China; 2 Center for Immune-related Diseases at Shanghai Institute of Immunology, Ruijin Hospital, Shanghai Jiao Tong University School of Medicine, Shanghai, 200025 China; 3 Department of Colorectal Surgery, Changhai Hospital, Naval Medical University, Shanghai, 200433 China; 4 Department of Anesthesiology, Ruijin Hospital, Shanghai Jiao Tong University School of Medicine, Shanghai, 200025 China; 5 Department of Medical Oncology, The 960th Hospital of the PLA Joint Logistice Support Force, Jinan, 250031 Shandong China; 6 Department of Radiation Oncology, LongHua Hospital Shanghai University of Traditional Chinese Medicine, Shanghai, 200032 China

**Keywords:** Tumour immunology, Tumour immunology, Neutrophils

## Abstract

Although recent research has established that gasdermin D (GSDMD), a factor that drives pyroptosis, is essential for cell death and inflammation, its involvement in cancer metastasis has yet to be elucidated. In this study, GSDMD was significantly increased in lung neutrophils at the metastatic stage from a murine orthotropic 4T1 breast cancer model. Moreover, the N terminal domain from cleaved GSDMD exhibited a positive correlation with increased mitochondrial reactive oxygen species (mROS) and serum high mobility group box 1 (HMGB-1) levels. Mechanistically, mROS inhibition significantly suppressed GSDMD-N oligomerization and pore formation. In addition, the activation of GSDMD significantly enhanced the formation of neutrophil extracellular traps (NETs) following treatment with Cathepsin C. Within a murine orthotopic breast cancer model using 4T1 cell line, the inhibition of GSDMD through the application of LDC7559 significantly attenuated the metastatic spread of breast cancer to the lung. In addition, knockout of GSDMD reduced lung metastasis in E0771 intravenous injection murine model. Furthermore, inhibition of GSDMD reduced the number of myeloid derived suppressor cells (MDSC) in the metastatic lung of breast cancer mouse model, while concurrently increasing both the percentage and total cell count of CD8^+^ T cells, suggesting that mitochondrial dysfunction-dependent GSDMD activation promotes tumor microenvironment immunosuppression and NETs. GSDMD represents a promising therapeutic target for mitigating the metastatic progression of breast cancer to the lung.

## Introduction

Breast cancer is among the most prevalent malignancies affecting women worldwide [1]. Although survival rates have markedly improved, the incidence of breast cancer continues to increase globally [2]. Over the past decade, the integration of genomic and transcriptomic data on an unprecedented scale has unveiled distinct subtypes of breast cancer and clonal evolutionary trajectories [3–5]. Recent advancements in high-resolution single-cell and spatial technologies have underscored the significance of the entire breast cancer ecosystem and its various cellular neighborhoods [6]. Thus, it has been established that breast carcinoma cells inhabit a complex environment that includes a diverse array of cell types, such as infiltrating immune cells, fibroblasts, endothelial cells, pericytes, adipocytes, and parenchymal cells. The immune tumor micro environment (TME) has garnered significant attention owing to recent advancements in cancer immunotherapy. Nonetheless, the efficacy of anti-tumor immunity is often compromised by the immunosuppressive characteristics of the TME in numerous breast cancer patients [7, 8]. In addition to CD8^+^ T cells, recent single-cell studies of breast cancer have elucidated distinct subsets of breast-resident γδ T cells, natural killer (NK), and CD4^+^ T cells [9].

Neutrophils constitute the second major myeloid population within the breast cancer microenvironment. An elevated circulating neutrophil-to-lymphocyte ratio has been identified as a significant biomarker of adverse outcomes in breast cancer, particularly in cases of triple-negative breast cancer (TNBC) [10]. Neutrophils respond to bacterial infections by generating neutrophil extracellular traps (NETs), which are intricate structures composed of DNA, histones, and granular enzymes. While NETs were initially observed exclusively at sites of bacterial infection, recent investigations have revealed their presence in various non-bacterial conditions, such as gout, autoimmune disorders, and COVID-19. Emerging research focused on the involvement of NETs in tumor metastasis and immune evasion. Notably, one study demonstrated NETs promote the migration and adhesion of human breast cancer cells through the Coiled-coil domain-containing protein 25 (CCDC25) gene [11]. Additionally, cathepsin C (CTSC) released by breast tumors has been implicated in facilitating breast-to-lung metastasis via NETs [12].

Gasdermin D (GSDMD) functions as a crucial pore-forming protein in the process of pyroptosis [13, 14]. Upon cleavage by caspase-1 or caspase-11, the N-terminal domain of GSDMD (GSDMD-N) is relocated to the plasma membrane. At this site, GSDMD-N undergoes oligomerization, resulting in the formation of pores in the membrane. This process ultimately induces cell lysis and triggers the release of pro-inflammatory cytokines, namely interleukin-1β (IL-1β) and interleukin-18 (IL-18) [15, 16]. Initially, GSDMD was thought to only induce pyroptosis of macrophages. However, several studies found that GSDMD can induce cell death in other intrinsic organs, such as lung epithelial cells, liver cells, and retinal pigment epithelial cells [17–19]. GSDMD exhibits a high level of expression in neutrophils and is integral to the processes of neutrophilic cell death as well as the formation of NETs [20]. Also, caspase-11 in neutrophils can induce NETs formation by cleaving GSDMD after gram-negative bacteria infection, thus promoting the killing effect on bacteria. Furthermore, neutrophil-specific elastase can cleave the 269th cysteine of GSDMD, thus promoting neutrophil death. GSDMD is known to interact with cardiolipin, a key component of the mitochondrial membrane. Upon activation, GSDMD can induce the release of mtDNA into the cytosol of endothelial cells through the formation of mitochondrial pores. Recent research has highlighted the essential role of GSDMD in several autoimmune diseases, such as familial Mediterranean fever, experimental autoimmune encephalomyelitis, and systemic lupus erythematosus. Nevertheless, its specific role in cancer immunology has yet to be determined.

This study sought to elucidate the physiological role of GSDMD in the pathogenesis of breast cancer and to characterize the regulatory mechanism by which mROS mediate pyroptosis in cancer metastasis. The results demonstrated that the activation of GSDMD in neutrophils is essential to produce pro-inflammatory cytokines and the formation of NETs, thereby promoting breast cancer metastasis.

## Results

### GSDMD is significantly upregulated in neutrophils from metastatic lung of triple negative breast cancer mouse model

**Fig. 1:**
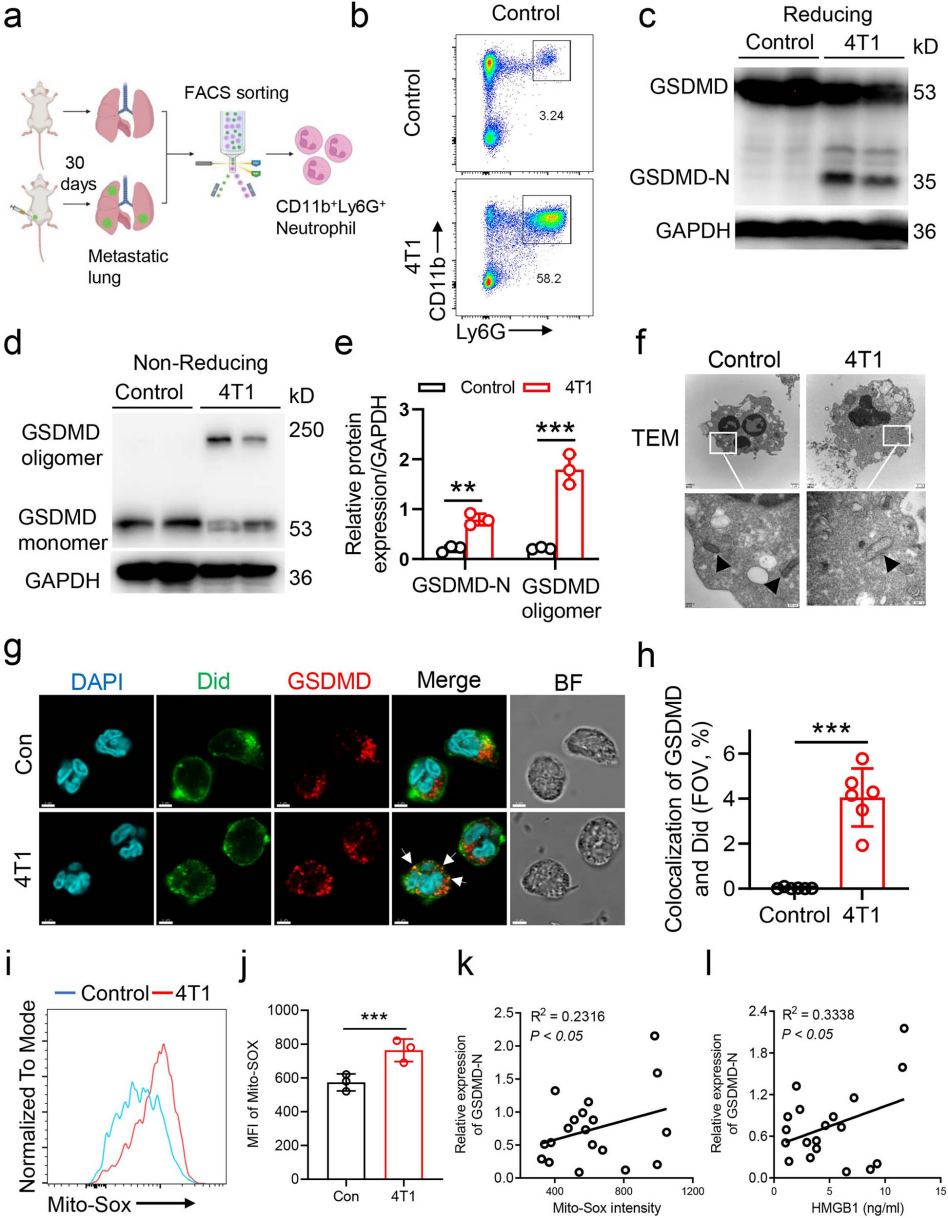
The upregulation of GSDMD and mROS in neutrophils from breast cancer metastatic mouse model. **a** The diagram of animal model. **b** Fluorescence-activated Cell Sorting analysis of lung neutrophils from control and 4T1 mice, cells were sorted by flow cytometry (CD11b^+^Ly6G^+^). **c** Immunoblot analysis of GSDMD and GSDND-N of lung neutrophils sorted from control and 4T1 mice. **d** Non-reducing immunoblot analysis of GSDMD monomer and oligomer of lung neutrophils sorted from control and 4T1 mice. **e** Quantification of GSDMD (the ratio of GSDMD-N to GAPDH in (**c**), the ratio of GSDMD oligomer to GAPDH in (**d**). n = 3. **f** Transmission electron microscope images of mitochondria in neutrophil from lung of control and 4T1 bearing mice. Scale bar, 200 nm. **g** Immunofluorescence staining of GSDMD in cytosol and cell membrane (Did staining) in lung neutrophils from control and 4T1 bearing mice. Scale bar, 3 μm. **h** Quantitative analysis of colocalization of GSDMD and Did. **i** Histograms of Mito-Sox fluorescence intensity in neutrophils from control and 4T1 bearing mice analyzed by flow cytometry. **j** Quantification of mean fluorescence intensity (MFI) of Mito-Sox as indicated in (**f**). n = 3. **k** Relative expression of GSDMD-N/GAPDH in bone marrow neutrophils is positively correlated with Mito-Sox intensity in lung neutrophils from 4T1 bearing mice. **l** Relative expression of GSDMD-N/GAPDH in lung neutrophils is positively correlated with serum HMGB1 from 4T1 bearing mice. Data are presented as means ± SEM. Pearson’s correlation analysis for (**k**, **l**). Significance was examined with two-tailed unpaired Student’s *t* test (**e**) or Student’s *t* test (**h**, **j**). ^**^P < 0.01, ^***^P < 0.001.

The alteration of the immune microenvironment is crucial for the initiation and advancement of breast cancer, especially breast cancer metastasis [21]. Neutrophils which are the predominant type of granulocytes found in peripheral blood, facilitate breast cancer metastasis through the production of neutrophil extracellular traps (NETs) [11, 22]. Herein, we evaluated the role of neutrophils and GSDMD-dependent NETs in the pathogenesis of breast cancer metastasis. Specifically, 4T1 cells were orthotopically implanted into the fourth left mammary fat pad. Neutrophils were then isolated from the metastatic lung at 30 days after 4T1 cell injection through fluorescence-activated cell sorting (Fig. 1a). The proportion of neutrophils in the lungs was significantly elevated in 4T1 mice when compared to the control group (Fig. 1b). However, the cleaved GSDMD-N and GSDMD oligomer were only detected in lung neutrophils from 4T1 mice (Fig. 1c–e). A transmission electron microscope (TEM) further investigated the mitochondria in lung neutrophils. TEM results showed that the lung neutrophils had intact mitochondria with bubbled round mitochondria and NETs formation (Fig. 1f). Immunofluorescence analysis revealed that GSDMD exhibited a diffuse expression pattern in the cytosolic region of neutrophils from control lungs, whereas it was observed to translocate to the plasma membrane in lung neutrophils derived from 4T1 mice (Fig. 1g, h). Mitochondrial reactive oxygen species (mROS) are integral to the oligomerization of GSDMD and the subsequent formation of pores [23]. Compared with control mice, the level of mROS was significantly elevated in lung neutrophils from 4T1 mice (Mito-Sox detection) (Fig. 1i, j). Meanwhile, the level of GSDMD-N was positively correlated with mROS (Fig. 1k). GSDMD-dependent pyroptosis is essential for high mobility group box 1 (HMGB-1) secretion. Further results showed that serum HMGB1 level from mouse breast cancer model was positively correlated with GSDMD-N expression (Fig. 1l). Mitochondrial quality control (MQC) is an integrated network that monitors mitochondrial integrity and serves as an intrinsic cellular protective mechanism. It is essential for maintaining mitochondrial homeostasis and function. MQC coordinates multiple processes—including mitochondrial biogenesis, fission, fusion, proteolytic degradation of mitochondrial proteins, and mitophagy—to collectively regulate and preserve mitochondrial homeostasis. The mRNA level of MFN1, MFN2, OPA1 and Fis1 were significantly reduced in lung neutrophils from 4T1 mice, while the level of TFAM was increased (Supplementary Fig. S1a). The immunofluorescence data showed that the intensity of Mito-tracker was significantly reduced in lung neutrophils from 4T1 mice (Supplementary Fig. S1b, c). In summary, the results indicate a significant upregulation of mROS and GSDMD in lung neutrophils obtained from the breast cancer mouse model.

### GSDMD inhibition significantly suppresses breast cancer progression to lung metastasis

**Fig. 2:**
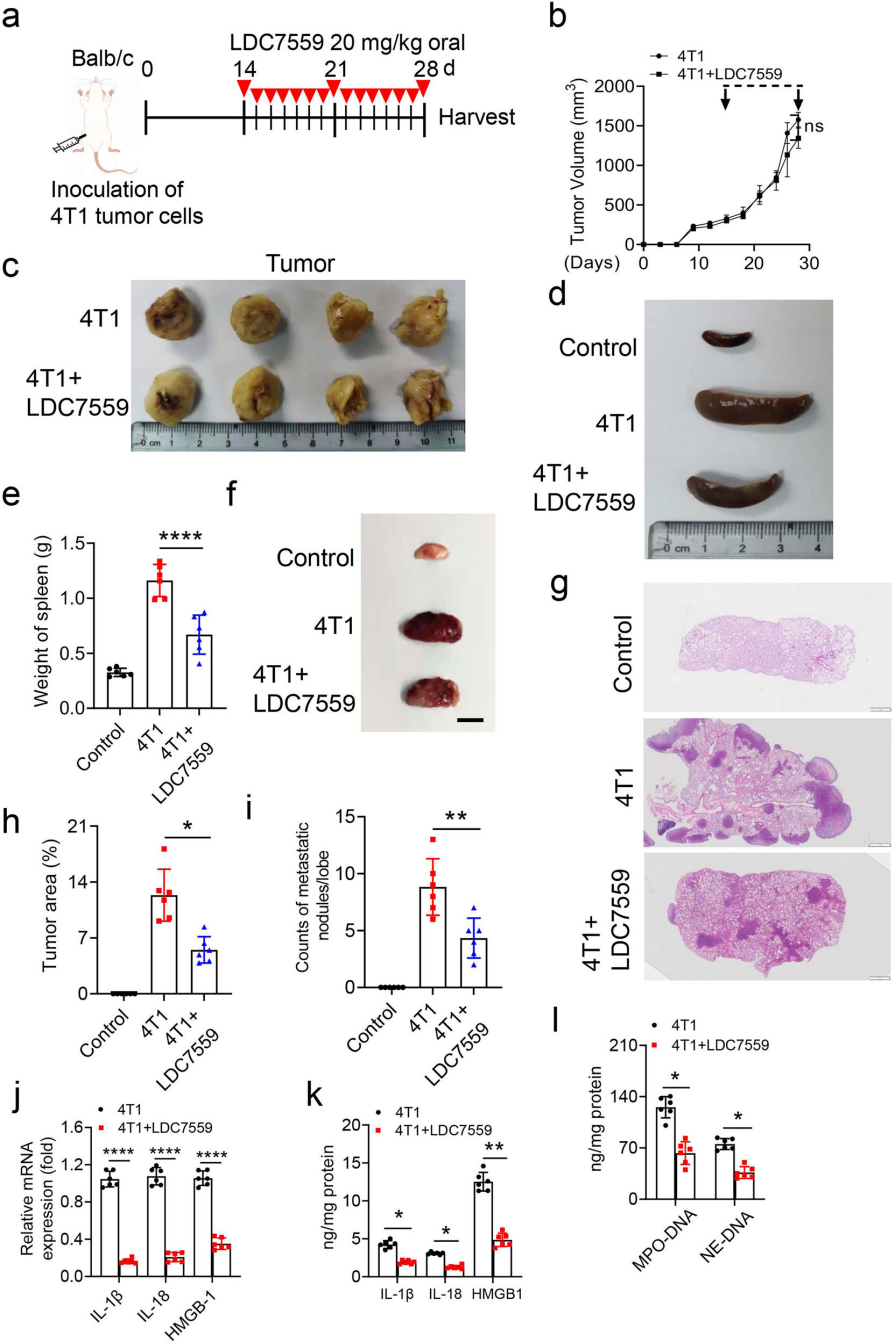
Inhibition of GSDMD by LDC7559 suppressed breast cancer to lung metastasis in 4T1 bearing mice. **a** Scheme of drug treatment strategy. 4T1 cells were injected into fourth mammalian pat of Balb/c mice for two weeks, and LDC7559 was performed daily for 2 weeks. **b** Quantitative analysis of tumor growth curve with and end-point tumor volume at day 28 in 4T1 and LDC7559 treated tumor model. Arrow in 14 days is the first time of LDC7559 treatment. **c** Photograph of excised solid tumor taken on day 28. **d** Photograph of spleen from 4T1 and LDC7559 treated 4T1 bearing mouse model extracted at day 28. **e** Quantitative analysis of the weight of spleen in control, 4T1 and LDC7559 treated 4T1 bearing mouse group. **f** Photographs of lungs from control, 4T1 and LDC7559 treated 4T1 bearing mouse model extracted at day 28. Scale bar, 1 cm. **g** Representative hematoxylin-eosin staining (H&E) staining of 4T1 metastasis tumor area in the lung tissue sections. Scale bar, 1 mm. **h** The 4T1 metastasis tumor area was calculated in (**g**). **j** qPCR analysis of IL-1β, IL-18 and HMGB-1 mRNA level in lung (GAPDH was used as an endogenous reference for qPCR). **k** ELISA analysis of IL-1β, IL-18 and HMGB-1 protein levels in lung from 4T1 and LDC7559 treated tumor model. **l** ELISA analysis of MPO-DNA and NE-DNA level in serum samples from 4T1 and LDC7559 treated tumor model. n = 6 mice. Data are presented as means ± SEM. Significance was examined with one-way ANOVA (**e**, **h**, **i**), or two-tailed unpaired Student’s *t* test (**b**, **j**, **k**, **l**). ns not significant, ^*^P < 0.05, ^**^P < 0.01, ^****^P *<* 0.0001.

A previous study showed that LDC7559 (GSDMD inhibitor) can efficiently inhibit phorbol-12-myristate-13-acetate (PMA)-induced NETs formation without inducing toxicity to peripheral blood mononuclear cells [24]. In this study, we assessed the impact of LDC7559 on tumor metastasis using 4T1 breast cancer cell line. LDC7559 was administered orally daily for an additional two weeks. As illustrated in Fig. 2a, tumor volume exhibited a significant increase after one week. Furthermore, LDC7559 did not exert a significant effect on tumor size during the period following the injection of 4T1 cells (Fig. 2b, c). Notably, treatment with LDC7559 resulted in a considerable reduction in spleen volume (Fig. 2d, e). To evaluate lung metastasis, the lungs were assessed through hematoxylin and eosin staining. The findings indicated that the degree of tumor metastasis was proportional to both the quantity and dimensions of metastatic nodules observed in 4T1 model. However, LDC7559 treatment markedly reversed these effects (Fig. 2f). Additionally, treatment with LDC7559 led to a marked decrease in lung metastasis in the 4T1 mouse model (Fig. 2g–i). The impact of LDC7559 on the immune response was analyzed through the assessment of pro-inflammatory cytokine expression levels in lung tissue. LDC7559 treatment led to a reduction in the transcriptional levels of pro-inflammatory cytokines, including IL-1β, IL-18, and HMGB1 (Fig. 2j). Concurrently, LDC7559 treatment also diminished these pro-inflammatory cytokines and HMGB1 levels (Fig. 2k). A prior study has reported the detection of NETs in serum. Herein, LDC7559 decreased the levels of serum MPO-DNA and NE-DNA (Fig. 2l). These results confirm that LDC7559 can suppress breast cancer to lung metastasis progression.

To further elucidate the role of GSDMD in breast tumor metastasis, we conducted a comparative analysis of disease pathogenesis in GSDMD-KO mice and their wild-type littermates with the injection of E0771 [25]. We firstly used an in-situ tumor model, in which E0771 was injected in the fourth left mammary fat pad (Supplementary Fig. S2a). The tumor volume and weight were not affected in *Gsdmd*^*−/−*^ mice after E0771 injection (Supplementary Fig. S2b–d). Unfortunately, we did not detect lung metastasis in this model (Supplementary Fig. S2e). We then explore the role of neutrophil-mediated tumor metastasis in *Gsdmd*^*−/−*^ mice. E0771 cells were intravenous injected (Supplementary Fig. S3a). After 2 weeks, we identified an increased lung metastasis after E0771 intravenous injection. More importantly, the metastatic nodules/lobe was significantly reduced in *Gsdmd*^*−/−*^ mice after E0771 inoculation (Supplementary Fig. S3b. The mRNA and protein level of IL-1β, IL-18 and HMGB-1 in lung were reduced in *Gsdmd*^*−/−*^ mice after E0771 inoculation (Supplementary Fig. S3c, d). In addition, serum level of MPO-DNA and NE-DNA were also blunted after E0771 inoculation (Supplementary Fig. S3e).

### GSDMD inhibition remodels immune cells in tumor microenvironment

**Fig. 3:**
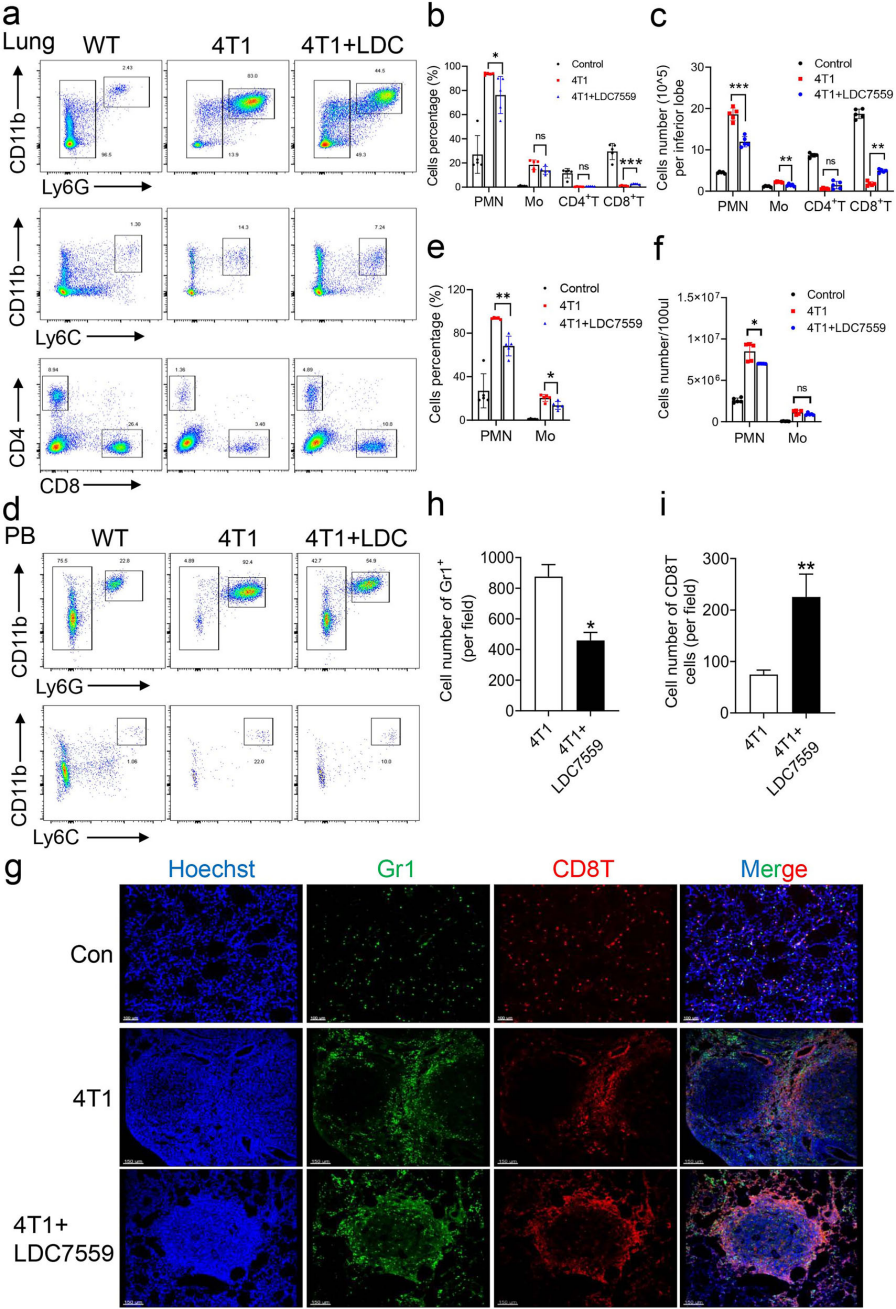
Inhibition of GSDMD by LDC7559 relieved immunosuppression in 4T1 bearing mice. **a** Flow cytometry analysis scheme of G-MDSC (CD45^+^CD11b^+^Ly6G^+^), M-MDSC (CD45^+^CD11b^+^Ly6C^+^), CD4^+^ T cells (CD45^+^CD4^+^) and CD8^+^ T cells (CD45^+^CD8^+^) in lung of indicated groups. **b** Quantification of the percentages of G-MDSC, M-MDSC, CD4^+^ T cells and CD8^+^ T cells in (**a**). **c** Quantification of absolute number of G-MDSC, M-MDSC, CD4^+^ T cells and CD8^+^ T cells in per lobe of lung in indicated groups. **d** Representative flow cytometric plots of peripheral blood G-MDSC and M-MDSC in wild type mice, 4T1-bearing mice, or LDC7559 treated 4T1-bearing mice. **e** Quantification of the percentages of G-MDSC and M-MDSC in (**d**). **f** Quantification of absolute number of G-MDSC and M-MDSC in (**d**). **g** Representative immunofluorescence analysis of Gr1 and CD8 in lung from wild type mice, 4T1-bearing mice, or LDC7559 treated 4T1-bearing mice. Scale bar, 100 μm. Cell numbers of Gr1^+^ cells (**h**) and CD8^+^ T cells (**i**) were calculated in (**g**). *n* = 5 mice. Data are presented as means ± SEM. Significance was examined with two-tailed unpaired Student’s *t* test (**b**, **c**, **e**, **f**) or Student’s *t* test (**h**, **i**). ns not significant, ^*^P < 0.05, ^**^P < 0.01, ^***^P < 0.001.

Immune cells (myeloid cells or lymphoid cells) play a crucial role in regulation of tumor immunity within the tumor immune microenvironment (TME) [26]. The TME has the capacity to reprogram neutrophils, enabling them to engage in cross-signaling with tumor cells and consequently alter the immune landscape of the tumors [27, 28]. Herein, the percentage of CD11b^+^Ly6C^+^ cells and CD11b^+^Ly6G^+^ cells significantly increased in the lung of breast cancer mouse model, while the percentage of T cells were reduced (Fig. 3a). A small molecule of LDC7559 was then used to inhibit GSDMD and phagocytic oxidative burst in neutrophils [28, 29]. LDC7559 treatment reduced the number of monocytes and neutrophils in lungs of breast cancer mouse model. Also, administration of LDC7559 led to a significant increase in the proportion of CD8^+^ T cells present in the lung tissue of the treatment group (Fig. 3a–c). Furthermore, LDC7559 treatment reduced the percentage of monocytes and neutrophils in peripheral blood (Fig. 3d–f). Immunofluorescence analysis revealed the presence of Gr1⁺ positively stained myeloid cells and CD8⁺ T cells within metastatic tumors. Treatment with LDC7559 resulted in a reduction of tumor-infiltrated myeloid cells while simultaneously increasing the levels of infiltrating CD8⁺ T cells in the TME (Fig. 3g–i). These results indicate that GSDMD inhibition can prevent breast cancer progression to lung metastasis by reshaping the TME.

### GSDMD inhibition suppresses hematopoiesis

**Fig. 4:**
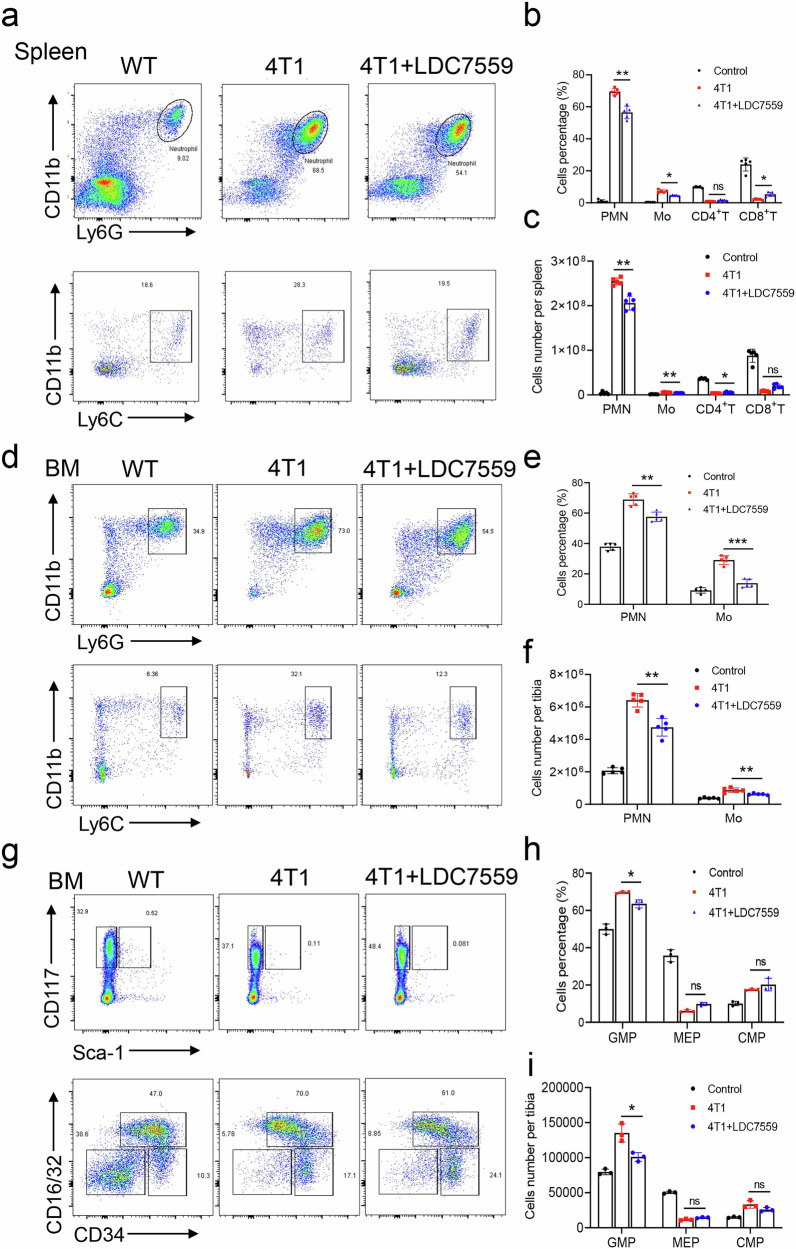
LDC7559 inhibits G-MDSC and M-MDSC expansion and hematopoiesis in bone marrow of breast cancer model. **a** FACS (Fluorescence-activated cell sorting) analysis of G-MDSC and M-MDSC in spleens of WT mice, 4T1-bearing mice or LDC7559 treated 4T1-bearing mice. **b** Quantification of the percentages of G-MDSC, M-MDSC, CD4^+^ T cells and CD8^+^ T cells in the spleen of indicated groups in (**a**). **c** Quantification of absolute cell number of G-MDSC, M-MDSC, CD4^+^ T cells and CD8^+^ T cells of whole spleen in WT, 4T1-bearing and LDC7559 treated 4T1-bearing mice. **d** FACS analysis of bone marrow G-MDSC and M-MDSC in wild type mice, 4T1 tumor bearing mice, or LDC7559 treated 4T1 tumor bearing mice. **e** Quantification of the frequency of G-MDSC and M-MDSC subsets in the bone marrow of indicated groups in (**d**). **f** Quantification of absolute cell number of G-MDSC and M-MDSC per tibia in WT, 4T1-bearing and LDC7559 treated 4T1-bearing mice. **g** FACS analysis of bone marrow lineage^-^c-Kit^+^Sca-1^+^ (LK), lineage^-^c-Kit^+^Sca-1^-^ cells (LSK), granulocyte-macrophage progenitors (GMP, lineage^-^c-Kit^+^Sca-1^+^CD34^+^CD16/32^+^), common myeloid progenitors (CMP, lineage^-^c-Kit^+^Sca-1^+^CD34^+^CD16/32^-^) and megakaryocyte-erythroid progenitors (MEP, lineage^-^c-Kit^+^Sca-1^-^CD34^-^CD16/32^-^) in WT, 4T1-bearing and LDC7559 treated 4T1-bearing mice. **h** Quantification of the percentages of LK, LSK, GMP CMP and MEP in (**g**). **i** Quantification of absolute cell number of LK, LSK, GMP CMP and MEP in (**g**). *n* = 5 mice (**b**, **c**, **e**, **f**). *n* = 3 mice (**h** and **i**). Data are presented as means ± SEM. Significance was examined with two-tailed unpaired Student’s *t* test (**b**, **c**, **e**, **f**, **h**, **i**). ns not significant, ^*^P < 0.05, ^**^P < 0.01, ^***^P < 0.001.

Invasive breast cancer generates immunosuppressive neutrophils by reprograming early myeloid differentiation in the bone marrow [30]. Also, bone metastasis in breast cancer can be prevented by modulating bone marrow hematopoietic lineage potential [31]. In this study, we evaluated the percentage of neutrophils isolated from the spleen and bone marrow to elucidate the specific role of neutrophils in breast cancer metastasis. The results indicated a notable increase in the percentage of neutrophils in the spleen, indicative of heightened migration from the bone marrow (Fig. 4a). However, treatment with LDC7559 led to a significant reduction in both the percentage and absolute count of neutrophils present in the spleen (Fig. 4b, c). Compared with the control, the percentage of bone marrow neutrophils significantly increased in 4T1 mice, indicating a strong effect on hematopoiesis (Fig. 4d–f). Further analyses showed that the population of GMP and CMP significantly increased in 4T1 mice while the level of MEP decreased. LDC7559 suppressed GMP levels through GSDMD inhibition. Notably, MEP and CMP cell populations were similar between the two groups (Fig. 4g–i). Taken together, these results demonstrate that GSDMD inhibition suppresses hematopoiesis in breast cancer mouse model.

### Inhibition of mitochondrial reactive oxygen species reduced GSDMD oligomerization and breast to lung metastasis

**Fig. 5:**
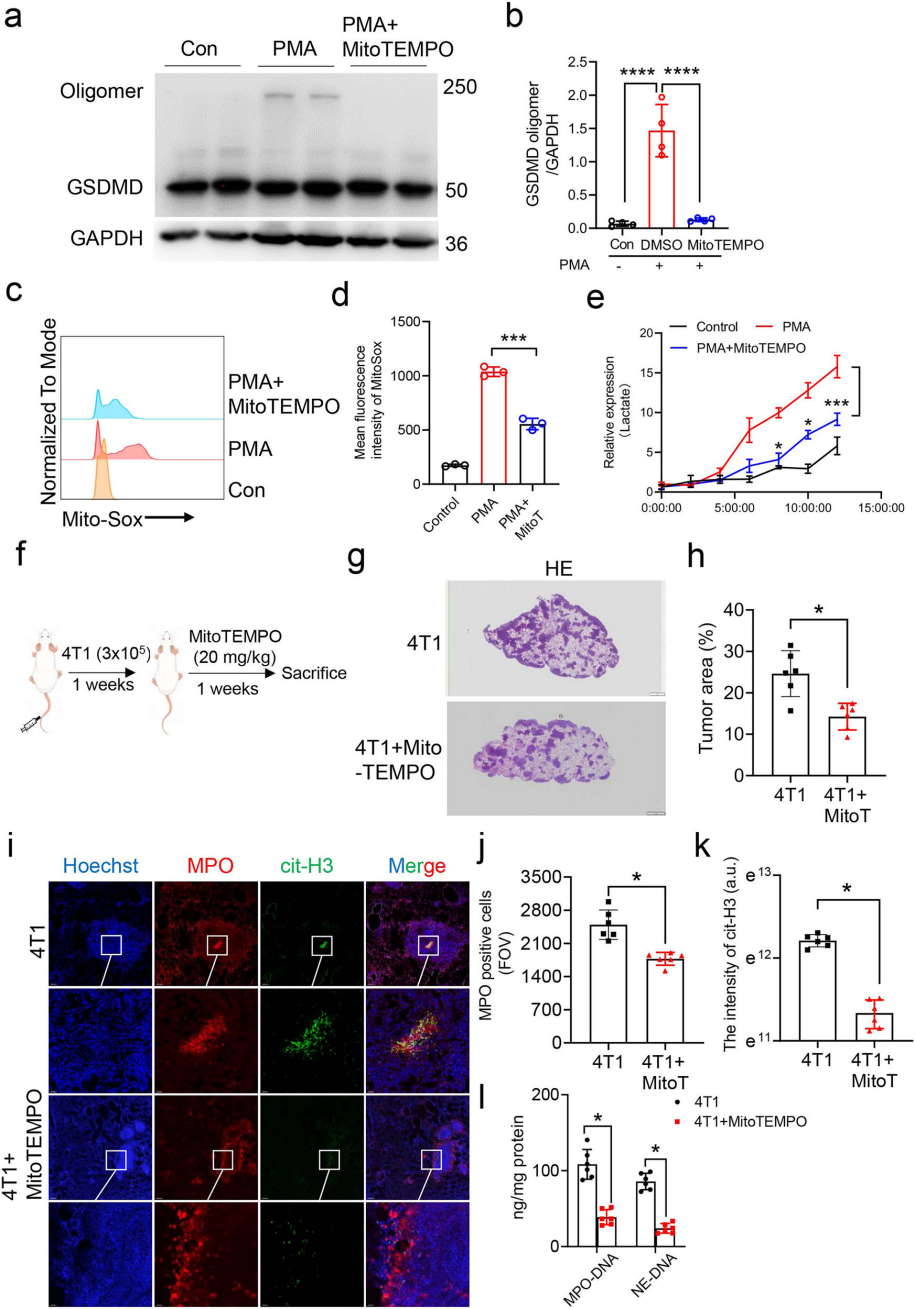
Inhibition of mROS by MitoTEMPO suppressed GSDMD oligomerization and breast cancer to lung metastasis. **a** Bone marrow neutrophils were isolated from balb/c mice. **b** Cells were pretreated with MitoTEMPO and treated with PMA for 12 h. Western blot images showing expression of GSDMD and GSDMD oligomer in bone marrow neutrophils from Control, PMA or PMA with MitoTEMPO pretreatment. **c** Flow cytometry analysis of Mito-Sox in bone marrow neutrophils after treatment with DMSO, PMA or PMA with MitoTEMPO pretreatment. **d** Quantification of MitoSox intensity on neutrophils as indicated in (**c**). *n* = 3. **e** ELISA results showing expression of LDH in the cultured medium from bone marrow neutrophils after DMSO, PMA or PMA with MitoTEMPO pretreatment. **f** Scheme of drug treatment strategy. 4T1 cells were intravenous injected into Balb/c mice for one weeks, and then MitoTEMPO treatment was performed daily for 1 weeks. **g** Representative hematoxylin-eosin staining (H&E) staining of 4T1 metastasis tumor area in the lung tissue sections. Scale bar, 1 mm. **h** The tumor metastatic area was calculated in (**g**). **g** Quantification of mean fluorescence intensity of MitoSox in (**f**). **i** Immunofluorescence of MPO and cit-H3 from 4T1 and 4T1 with MitoTEMPO treatment mice. Scale bar, 200 μm. **j** Quantitative analysis of MPO positive cells in field of view from 4T1 and 4T1 with MitoTEMPO treatment mice. **k** Quantitative analysis of citH3 intensity in field of view from 4T1 and 4T1 with MitoTEMPO treatment mice. **l** ELISA analysis of MPO-DNA and NE-DNA level in serum samples from 4T1 and MitoTEMPO treated mouse tumor model. *n* = 6 mice. Data are presented as means ± SEM. Significance was examined with Student’s *t* test (**h**, **j**, **k**) or two-tailed unpaired Student’s *t* test (**b**, **l**). ^*^P < 0.05; ^***^P < 0.001;^****^P < 0.0001.

mROS facilitates GSDMD oligomerization, leading to pore formation and subsequent pyroptosis in macrophages [23]. Herein, the role of mROS in GSDMD oligomerization in neutrophils was also investigated. To mitigate the production of mROS, we utilized MitoTEMPO, a selective superoxide scavenger targeted at mitochondria. Treatment with PMA leading to a significant enhancement of GSDMD oligomerization, whereas the application of MitoTEMPO effectively inhibited this oligomerization process (Fig. 5a, b). Indeed, PMA treatment significantly increased the intensity of Mito-Sox, while MitoTEMPO suppressed the intensity (Fig. 5c, d). Lactate dehydrogenase (LDH) was a widely used method to detect cell death including pyroptosis [32]. Furthermore, PMA treatment increased LDH levels, while MitoTEMPO decreased LDH levels (Fig. 5e). 4T1 cells were injected through the veil tail (Fig. 5f). After 2 weeks, we identified an increased lung metastasis after 4T1 intravenous injection. MitoTEMPO (a mitochondria-specific superoxide scavenger) was used to inhibit mROS production. More importantly, the metastatic nodules/lobe was significantly reduced after MitoTEMPO treatment (Fig. 5g, h). We also detected NETs by detecting the co-expression of MPO and cit-H3. The immunofluorescence data showed a significant increased positive staining of MPO and cit-H3 in 4T1 mice, which was reduced after MitoTEMPO treatment (Fig. 5i–k). In addition, serum level of MPO-DNA and NE-DNA in 4T1 model were also blunted after MitoTEMPO treatment (Fig. 5l). We further used mROS inducer rotenone, to determine whether artificially increasing mROS levels can directly promote GSDMD activation in neutrophils. In Supplemental Fig. 4, the cleaved GSDMD-N was significantly increased after Rotenone treatment, while this increase was suppressed by MitoTEMPO (Supplementary Fig. S4a, b). Collectively, the data indicated that inhibition of mROS suppressed GSDMD oligomerizing and NETs, thus reduced breast to lung metastasis.

### mROS and GSDMD promote NETs release after CTSC treatment

**Fig. 6:**
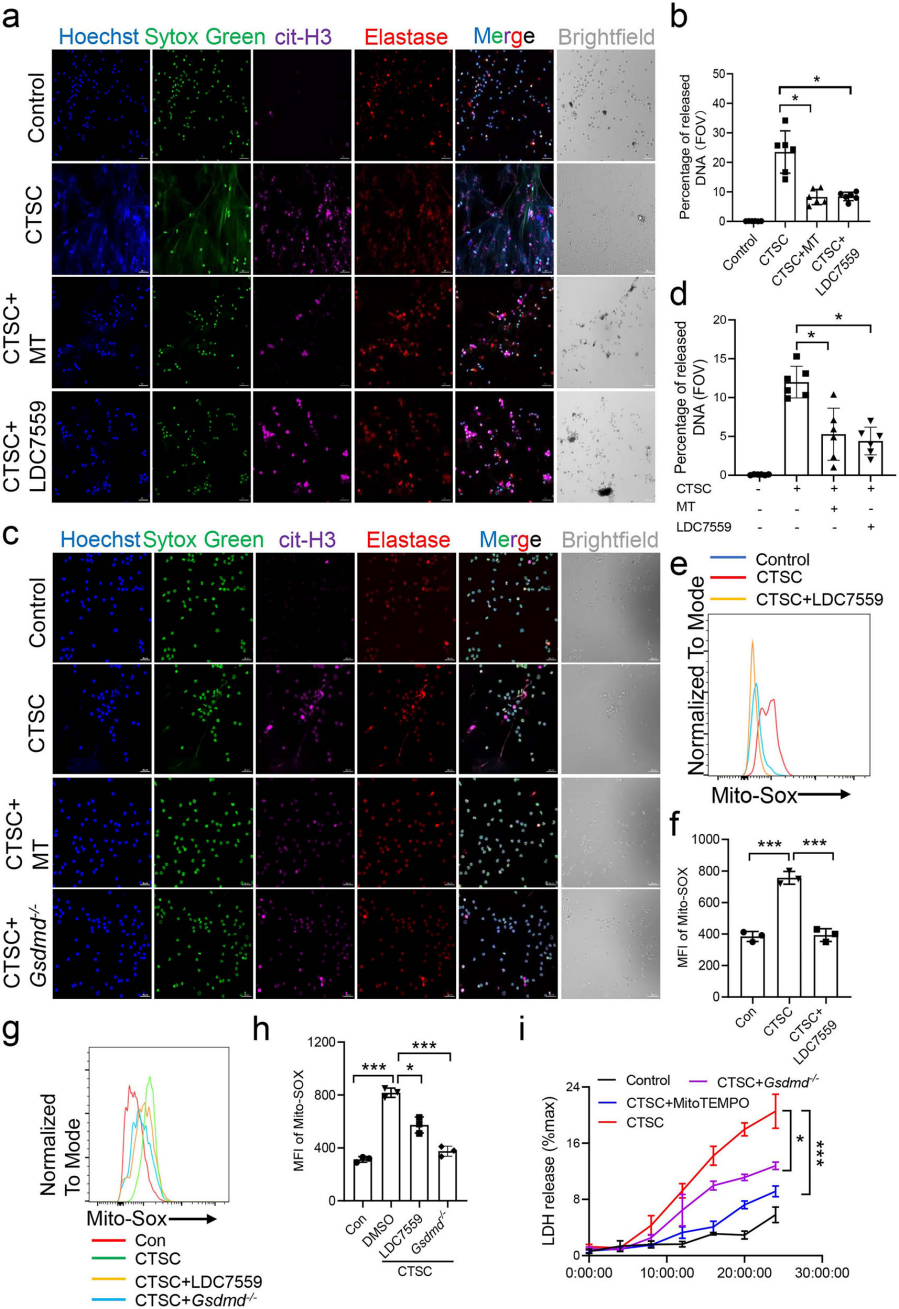
mROS inhibition or GSDMD knockout suppressed NETs in bone marrow neutrophils after CTSC treatment. **a** Immunofluorescence staining of cit-H3 (Red), Sytox Green (Green), Elastase (magenta) and Hoechst (Blue) in peripheral blood neutrophils from healthy volunteers. Cells were treated with DMSO, CTSC, CTSC + MitoTEMPO (MT) or CTSC + LDC7559. Scale bar, 100 μm. **b** Quantification of the percentage of released DNA per field of view from indicated groups in (**a**). **c** Immunofluorescence staining of cit-H3 (Red), Sytox Green (Green), Elastase (magenta) and Hoechst (Blue) in bone marrow neutrophils from WT and *Gsdmd*^*−/−*^ mice. Cells were treated with DMSO or CTSC. Scale bar, 30 μm. **d** Quantification of the percentage of released DNA per field of view from indicated groups in (**c**). **e** Representative histograms of Mito-Sox in peripheral blood neutrophils from healthy volunteers. Cells were treated with DMSO, CTSC or CTSC+LDC7559. **f** Quantification of Mito-Sox intensity from indicated groups in (**e**). **g** Representative histograms of Mito-Sox in bone marrow neutrophils from WT or *Gsdmd*^*−/−*^ mice. Cells were treated with DMSO, CTSC or CTSC + LDC7559. **h** Quantification of Mito-Sox intensity from indicated groups in (**g**). **i** Quantification of LDH released into the supernatant from bone marrow neutrophils after DMSO, CTSC or CTSC with MitoTEMPO pretreatment. Data are presented as means ± SEM. Significance was examined with one-way ANOVA (**b**, **d**, **f**, **h**), two-tailed unpaired Student’s *t* test (**i**). MFI mean fluorescence intensity. ^*^P < 0.05, ^***^P < 0.001.

The role of mROS/GSDMD pathway in mediating DNA release in NETs was also evaluated. Neutrophils were isolated from healthy volunteers (HVs), and cultured with Cathepsin C (CTSC). LDC7559 is an effective inhibitor of GSDMD [24, 29]. CTSC treatment caused excessive NETs formation, while MitoTEMPO or LDC7559 treated significantly suppressed DNA release (Fig. 6a, b). The involvement of the mROS/GSDMD pathway in the formation of NETs by bone marrow neutrophils in mice was also examined. Immunofluorescent staining demonstrated that treatment with CTSC prompted the release of NETs from wild-type neutrophils, whereas the application of MitoTEMPO significantly inhibited this release (Fig. 6c, d). Bone marrow neutrophils were isolated from *Gsdmd*^*−/−*^ mice to further confirm the role of GSDMD in CTSC-induced NETs. Also, CTSC-induced NETs release was significantly suppressed in neutrophils from *Gsdmd*^*−/−*^ mice (Fig. 6c, d), suggesting that mROS/GSDMD promotes NETs release after CTSC treatment.

The effect of GSDMD inhibition on mROS level was then assessed. The intensity of Mito-Sox in peripheral blood neutrophils from HVs was significantly increased after CTSC treatment, while LDC7559 treatment decreased the level of Mito-Sox (Fig. 6e, f). The role of GSDMD on mROS production in bone marrow neutrophils from WT mice was also investigated. Flow cytometry analysis indicated that treatment with CTSC significantly elevated Mito-Sox levels in bone marrow neutrophils. However, the level of Mito-Sox was suppressed in *Gsdmd*^*−/−*^ mice or after LDC7559 treatment (Fig. 6g, h). Furthermore, CTSC treatment increased LDH levels, while MitoTEMPO or GSDMD deficiency decreased LDH levels (Fig. 6i). Collectively, these results indicate that mROS and GSDMD promotes NETs after CTSC treatment.

### NETs promote breast cell epithelial-mesenchymal transition and proliferation

**Fig. 7:**
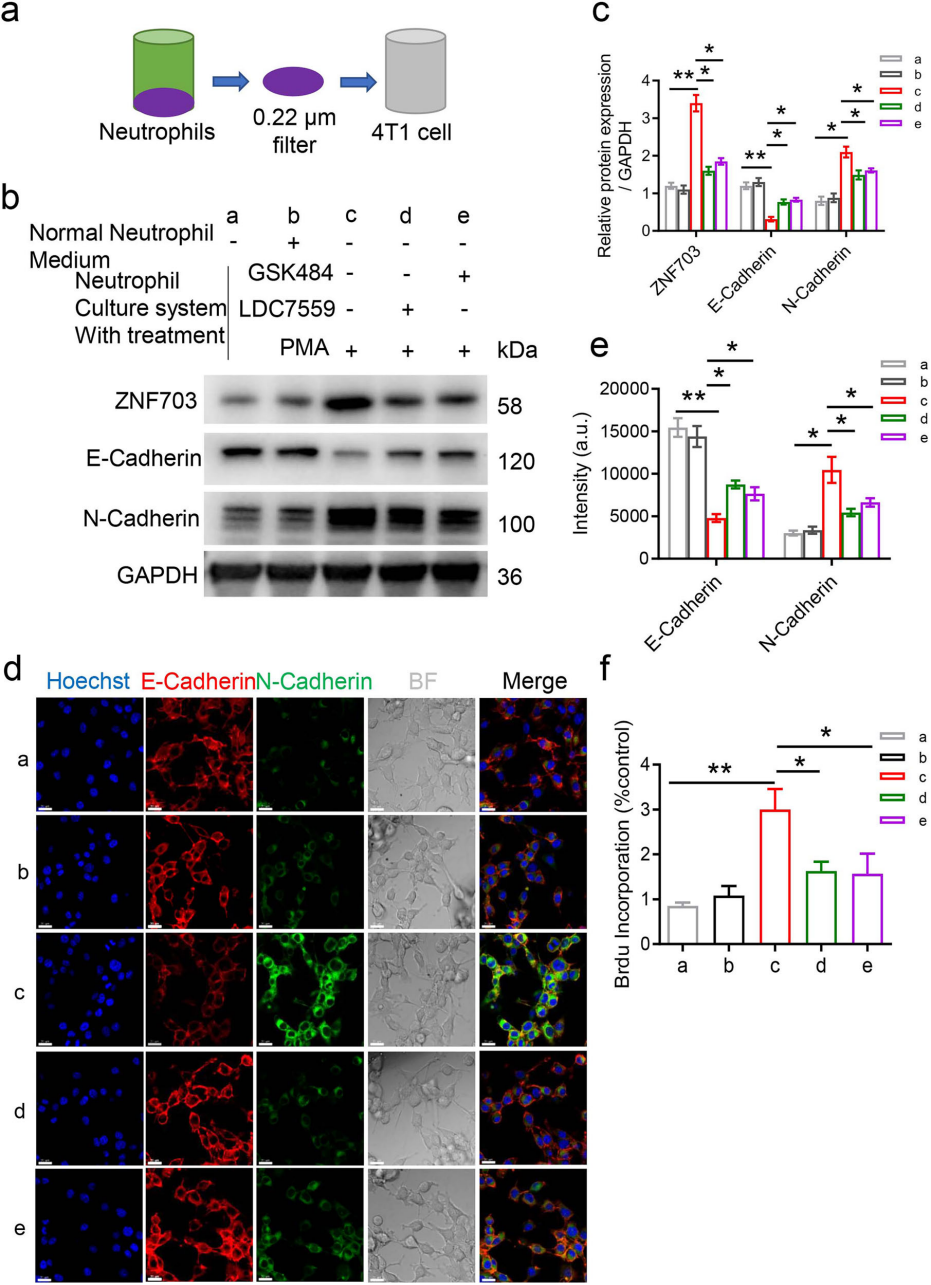
Inhibition of NETs suppressed breast cancer cell epithelial-mesenchymal transition and proliferation. **a** The scheme of neutrophils and 4T1 cells culture system. The medium from PMA-treated neutrophils was filtered by 0.22 μm strainer and then added to culture 4T1 cells. **b** Immunoblot analysis of ZNF703, E-cadherin and N-cadherin expression in 4T1 cells treated in different groups: **a** 4T1 cells in the normal cultured system. **b** The medium of 4T1 cells were replaced by neutrophil culture medium. **c** The medium of 4T1 cells were replaced by PMA-induced neutrophil cultured medium. **d** The medium of 4T1 cells were replaced by PMA with LDC7559 pretreatment-induced neutrophil cultured medium. **e** The medium of 4T1 cells were replaced by PMA with GSK484 pretreatment-induced neutrophil cultured medium. **c** Quantification of ZNF703, E-Cadherin and N-cadherin expression from indicated groups in (**b**). **d** Immunofluorescence staining of E-Cadherin and N-Cadherin of 4T1 cells after indicated treatment in (**b**). Scale bar, 20 μm. **e** Quantitative analysis of the fluorescence intensity of E-cadherin and N-cadherin in (**d**). **f** Quantitative analysis of BrdU incorporation of 4T1 cells from the indicated groups in (**b**). Data are presented as means ± SEM. Significance was examined with two-tailed unpaired Student’s *t* test (**c**, **e**) or one-way ANOVA (**f**). ^*^P < 0.05, ^**^P < 0.01.

Myeloid cell-induced inflammation and epithelial-mesenchymal transition (EMT) play a key role in breast cancer metastasis [33, 34]. In this study, the interaction between these two subsets of cells was investigated. Bone marrow neutrophils were isolated from WT mice. NETs were induced using PMA, and then the cultured medium was filtered through a 0.22 μm strainer. The medium was then added to the cultured system with 4T1 cells (Fig. 7a). EMT was not detected in 4T1 cells in the normal cultured system (indicated as “a”) or neutrophil culture medium without stimulation (indicated as “b”). Nevertheless, following PMA treatment, the replacement of the cultured medium of 4T1 cells with neutrophil-cultured medium resulted in a significant increase in the levels of ZNF703 and N-Cadherin. (indicated as “c”), while the level of E-cadherin decreased (compared with control) (Fig. 7b, c). Furthermore, PAD4 inhibitor GSK484 or LDC7559 treatment inhibited EMT activation (Fig. 7b, c). Furthermore, treatment with CTSC resulted in a significant reduction in the intensity of E-Cadherin while simultaneously increasing the levels of N-Cadherin in the neutrophil culture medium. However, treatment with the PAD4 inhibitors GSK484 or LDC7559 led to an increase in E-Cadherin levels and a concomitant decrease in N-Cadherin levels (Fig. 7d, e). CTSC treatment also increased the proliferation of 4T1 cells in a neutrophil culture medium. PAD4 inhibitor GSK484 or LDC7559 treatment decreased the level of BrdU (Fig. 7f). Collectively, these results indicate that GSDMD-dependent NETs promote breast cancer cell EMT and proliferation.

### Cellular constitution in lung of metastatic breast tumor lesions

**Fig. 8:**
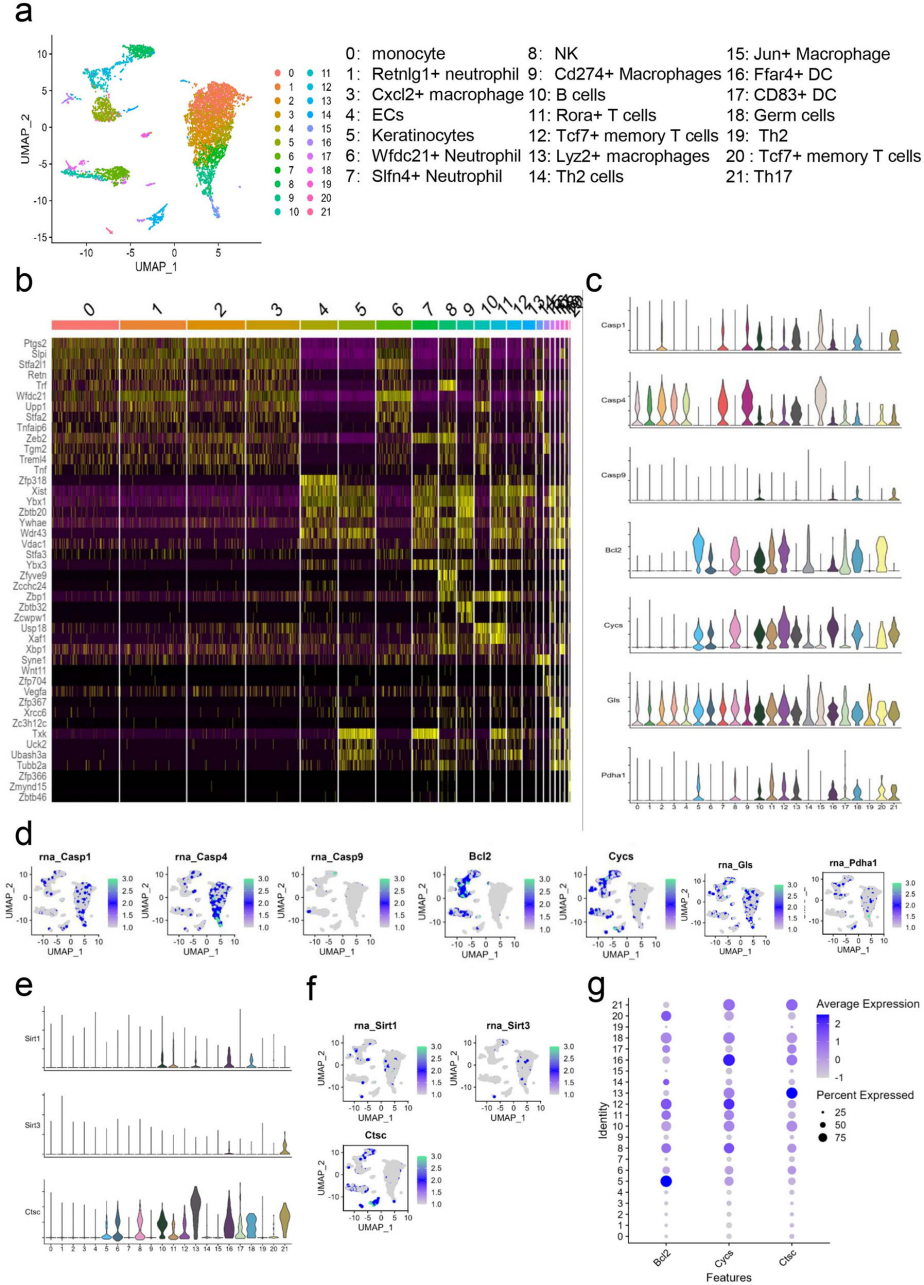
Cellular constitution and cell death-related genes on immune cells in TNBC. **a** UMAP plot illustrating all CD45^+^ cells in TNBC mouse models from GEO dataset. The 22 cell populations identified via transcriptional profiles indicated by different colors. **b** The heat map showing relative gene expression profile of genes in the top2 per cell in each cluster. **c**, **e** Violin plots showing the expression of Casp1, Casp4, Casp9, Bcl2, Cycs, Gls and Pdha1 based on cluster ID. **d**, **g** Feature plots displaying expression profile of genes mentioned in the above 22 clusters. **f** Dotplot showing the average expression and expressed percentage of Bcl2, Cycs and Ctsc in 0–21 clusters.

To investigate the cellular composition of triple-negative breast cancer (TNBC), we extracted TNBC data from GEO dataset, which includes CD45^+^ cells from mouse lungs with metastatic mammary cancer, as well as single-cell RNA sequencing (scRNA-seq) analysis data consisting of 22 cell subgroups. These subgroups mainly consist of nine cell types that were identified by unbiased clustering of cells based on gene expression profiles and canonical markers using uniform manifold approximation and projection (UMAP) and t-distributed stochastic neighbor embedding (t-SNE) analyses (Fig. 8a, b). In particular, the TNBC composition was as follows: (1) monocytes highly expressing *Ccl6, Hmgb2*, and *Retnlg*; (2) (6) and (7), neutrophils with high expression of *Slfn4, Fchsbl2*, and *Isg15*; (3) (9) (13) and (15), macrophages characterized with *Cst3, Cd274*, and *Lyz2*; (4), endothelial cells (ECs) highly expressing *Slfn4, Slpi*, and *Wfdc21*; (5), keratinocytes with high expression of *Nsa2, Tubb5*, and *mt-Co2*, (8) natural killer cells (NK) characterized by *Cd7, Serpinb6b*, and *Fyn*; (10), B cells specifically expressing *Ms4a1, Cd79a*, and *Cd79b*; (11) (12) (14) and (19-21), T cells expressing *Cd3g, Tcf7, Gata3* and *Il7r*; (16) and (17), dendritic cells (DCs) expressing *Ffar4, Mef2c*, and *Siglecg* (Fig. 8b). The expression profiles of key genes within the various cell populations were thoroughly analyzed. Among the eleven identified subclusters, the top 20 differentially expressed genes (DEGs) were determined for the seven predominant subclusters. A comprehensive discussion of these findings will be presented in the following sections. To further explore the expression of cell death-related genes, such as pyroptosis [caspase-1, caspase-4 and caspase-9]; apoptosis [(B-cell lymphoma-2 (Bcl2), cytochrome C (Cycs)]; and cuproptosis [glutaminas (Gls), pyruvate dehydrogenase complex (Pdha1)]. Through Seurat analysis, we found that Casp1 and Casp4 were highly expressed on Th2 cells, whereas *Cd27, Trat1* and *Gata3* were highly expressed (Fig. 8c, d). Bcl2 and Cycs were highly expressed on keratinocytes, NK, B cells and T cells (Fig. 8c, d). Similarly, cuproptosis-related genes were highly expressed on keratinocytes and T cells (Fig. 8c, d). Finally, we investigated whether epigenetic modification participates in the progression of TNBC. Data analysis showed that Sirt1 was expressed on dendritic cells. In contrast, elevated expression levels of cathepsin C (Ctsc) were observed in neutrophils, macrophages, and T cells (Fig. 8e, f). Dotplot showing the average expression and expressed percentage of Bcl2, Cycs and Ctsc in 0–21 clusters (Fig. 8g).

## Discussion

NETs are consisting of complex networks of proteins and DNA from neutrophils, which regulates the physiology and pathology of various immune inflammatory diseases, thrombosis and cancer [35]. Recent investigations have revealed the presence of NETs in the lungs of patients with breast cancer, and the formation of these NETs has been positively correlated with the metastasis of breast cancer [11, 12, 36]. While NETs represent a potentially promising therapeutic target, the precise mechanisms underlying the formation of NETs in the context of breast cancer metastasis, as well as the potential for their inhibition through small molecules, remain to be elucidated. Here, we identified that GSDMD mediated the effects of NETs on the mechanism of breast cancer metastasis. The GSDMD inhibitor significantly reduced CTST-induced NETs and mROS level. Furthermore, GSDMD inhibition remodeled the tumor micro-immune environment and hematopoiesis in bone marrow. These data suggested that GSDMD mediated the innate immune cell response in breast cancer metastasis.

NETs are structures composed of decondensed DNA that serve to capture and eliminate pathogens during infections. However, emerging evidence has identified a distinct class of NETs that may facilitate tumor growth, enhance cancer dissemination, and provide protective mechanisms against immune system attacks [37]. Our research demonstrates that inhibition of GSDMD using LDC7559 leads to a significant reduction in serum DNA complex, which effectively reduces the spread of breast cancer to the lungs in a murine model. In addition, a recent investigation has revealed that NETs are involved in the metastasis of gastric cancer in patients experiencing postoperative abdominal infections [38]. Consequently, it is essential to investigate the function of GSDMD-dependent NETs across various cancer types. Our in vitro findings indicate that the formation of NETs following treatment with CTSC is substantially impaired in neutrophils deficient in GSDMD. NETs consist of extracellular DNA fibers interwoven with histones and proteins from neutrophil granules, such as neutrophil elastase (NE), myeloperoxidase (MPO), and cathepsin G [39]. NET-associated DNA has been implicated in promoting cancer cell chemotaxis and facilitating the proteolytic remodeling of the extracellular matrix, which can activate dormant cancer cells, ultimately leading to breast cancer cell metastasis to the lungs [11, 40]. While we have validated the role of GSDMD in NET formation, the specific influence of GSDMD on breast cancer biology has not been thoroughly investigated in vitro. Further studies are warranted to explore the effects of CTSC-induced NETs on breast cancer cell proliferation, migration, and the epithelial-mesenchymal transition process.

This study shows that EMT of breast cancer cells did not occur after treatment with normal neutrophil medium. The results of our study indicate that the addition of neutrophils to PMA for 12 h significantly promotes the EMT of breast cancer cells in the neutrophil culture medium. Previous research has suggested that the formation of NETs occurs following PMA treatment [41]. Thus, we speculate that compounds from the NETs in the neutrophil culture medium activated EMT. Similarly, compounds in NETs such as MMP9, elastase, HMGB1 influenced the EMT in cancer cells [42]. Furthermore, the EMT of breast cancer cells was suppressed by the PAD4 inhibitor GSK484 or GSDMD inhibition LDC7559. These results indicated that NETs have important roles in breast cancer cells EMT and metastasis. Recently, the crosstalk between innate immune cells and breast cancer cells was reported to promote breast cancer metastasis [43–46]. It was observed that the formation of NETs is an essential effector promoting breast cancer cells metastasis [47].

Numerous potential therapeutic targets, including Ak strain transforming factors, cyclin-dependent kinases, poly (ADP-ribose) polymerase, and various growth factors, are currently being evaluated in clinical trials for breast cancer [48]. However, the available targeted therapies have not yet demonstrated significant therapeutic efficacy against triple-negative breast cancer (TNBC). Recently, immunotherapy has emerged as a promising treatment modality, and the U.S. Food and Drug Administration (FDA) granting approval to several immune checkpoint inhibitors for use in conjunction with chemotherapy in the management of TNBC [49]. Our in vivo data from in 4T1 mice further demonstrated that the NETs remodeled proinflammatory factors and recued CD8^+^ T cells activation in breast cancer cells, and these effects were reduced following GSDMD inhibition by LDC7559. Another study found that breast cancer cells are capable of internalizing NETs, and presenting arthritogenic peptides to T cells, thereby triggering systemic autoimmune and promoting tumor metastasis [50]. Our flow cytometry analysis revealed an increase in monocyte populations and a corresponding decrease in CD8^+^ T cells within the lungs following treatment with LDC7559. Additionally, confocal microscopy results indicated a reduction in CD68 expression and an elevation in CD8^+^ T cell populations in the breast tumor microenvironment in the lungs after LDC7559 administration. Thus, these observations suggest that the suppression of GSDMD may play a crucial role in enhancing the activation of immune cells, particularly CD8^+^ T cells. Recent research has also indicated that disulfiram (DSF), another inhibitor of GSDMD, may strengthen T-cell-mediated anti-tumor immunity by directly activating LCK-mediated T cell receptor (TCR) signaling pathways. The interaction between immune cells and breast cancer cells is highly complex, highlighting the urgent need for further investigations into the contributions of neutrophils, CD8^+^ T cells, and breast cancer cells within the tumor microenvironment.

GSDMD, a recently identified factor that drives pyroptosis, has been recognized for its critical involvement in infectious diseases, particularly sepsis [19, 51]. Analysis using Western blotting demonstrated that the GSDMD-N terminal was present solely in lung neutrophils sourced from 4T1 mice. Given that the GSDMD-N terminal is a reliable marker of pyroptosis [13], our results indicated that pyroptosis of neutrophils occurs in the lung during breast cancer metastasis. Previous studies found that GSDMD is both upregulated and activated in neutrophils [52]. One of critical functions of GSDMD is to facilitate pyroptosis pore, which promotes IL-1β secretion [53]. In a murine model of breast cancer, IL-1β was identified in the serum, and its expression levels demonstrated a positive correlation with those of GSDMD-N. This correlation implies that GSDMD is instrumental in mediating the effects of IL-1β within the breast cancer context in this model. Additionally, the cleavage of GSDMD occurs through both the canonical and non-canonical inflammasome pathways [54]. Caspase-1 serves as the effector enzyme within the canonical inflammasome, while caspase-11 functions as the effector enzyme in the non-canonical inflammasome. Future research should focus on elucidating caspase-1 and caspase-11 activation in neutrophils derived from both breast cancer patients and murine models. Additionally, further investigation should examine the exact mechanism underlying the activation and cleavage of GSDMD after CTSC treatment.

Earlier research has indicated that the murine breast cancer model induced by 4T1 is a widely used model for TNBC [55, 56]. In such models, GSDMD inhibition substantially reduced the release of pro-inflammatory cytokines, specifically IL-1β and IL-18, as well as HMGB1. The release of these inflammatory cytokines via GSDMD is recognized for its critical role in promoting the secretion of growth factors and the production of matrix metalloproteinases (MMPs) [57, 58]. Besides GSDMD is vital not only for the progression of breast cancer metastasis through GSDMD/NETs but also for the spread of breast cancer to the lungs by modulating the production of pro-inflammatory cytokines and MMPs in neutrophils. Our findings together illuminate a novel mechanism by which mitochondrial reactive oxygen species (mROS) initiate GSDMD-dependent NETs within the context of breast cancer metastasis. Furthermore, these insights may guide the development of NET inhibitors and strategies aimed at altering the interactions between neutrophils and breast cancer cells, identifying the mROS/GSDMD pathway as a promising therapeutic target for addressing breast cancer metastasis.

## Materials and methods

### Materials

Reagents and buffers utilized for molecular and cellular biology were sourced from Sangon Biotech (Shanghai). Dulbecco’s Modified Eagle’s Medium (DMEM), RPMI medium, and diamidino-2-phenylindole (DAPI) were procured from Sigma Millipore. The BCA Protein Assay Kit was obtained from Beyotime Biotechnology (P0010 Shanghai, China). Fetal bovine serum (FBS) was acquired from GIBCO (Sigma Millipore). Rotenone was obtained from MedChemExpress (HY-B1756). The MitoTracker™ Dyes for Mitochondria Labeling was acquired from ThermoFisher (M22426). Antibodies, including ZNF703 (ab219800), N-Cadherin (ab34710), Snail (ab2413), α-SMA (ab7817), and Anti-Histone H3 (ab5103) were obtained from Abcam. β-actin antibody (60008-1-Ig) and FITC anti-mouse CD68 Antibody (137005) were acquired from Proteintech and Biolegend, respectively. The PE-anti-mouse CD8a antibody and the APC-Rat anti-mouse/human CD11b antibody were acquired from BioLegend (San Diego, United States), with catalog numbers 100707 and 101212, respectively. Flow cytometry antibodies (PB-Rat anti-mouse Ly6G (Cat#127626, Biolegend), PE-anti-mouse Ly6C (Cat#128008, Biolegend), BV786-anti-mouse CD8a (Cat#100750, Biolegend), APC-anti-mouse CD11b (Cat#101212, Biolegend), Bv421 anti-mouse CD4 (Cat#100544, Biolegend) Biotin-TER-119/Erythroid Cells (Cat# 116204, Biolegend), Biotin-CD11b (Cat#101204, Biolegend), Biotin-Gr-1 (Cat#108404, Biolegend), Biotin-CD3ε (Cat#100304, Biolegend), Biotin-CD45R/B220 (Cat#103204, Biolegend), Biotin-IL-7Rα (Cat#135006, Biolegend) and Percp-Cy5.5-Streptavidin (Cat#405214, Biolegend) antibodies) were used for lineage staining. PB-Sca-1 (Cat#108120, Biolegend), APC-CD117 (Cat#105812, Biolegend), PE-CD34 (Cat#152204, Biolegend) and FITC-CD16/32 (Cat#101306, Biolegend) was used for HSPC staining.

### Animals

Balb/c mice were sourced from the SLAC Animal Center in Shanghai, China, ensuring that the selected specimens were of appropriate age and sex for the experimental procedures. Following acquisition, the mice were randomly distributed into distinct experimental groups. All animal studies adhered to the ethical guidelines set forth by the Laboratory Animal Administrative Committee of Shanghai Fourth People’s Hospital (approval ID: TJBH11924103). The mice were housed in a specific-pathogen-free (SPF) environment at Tongji University, maintained under controlled conditions with a 12-hour light/dark cycle, a temperature range of 21–22 °C, and humidity levels of 40–70%. Efforts were made to minimize any potential pain and discomfort in compliance with established animal welfare standards.

### Neutrophil isolation in a mouse model

Neutrophils were extracted from the bone marrow located within the tibias and femurs through the application of Hank’s Balanced Salt Solution (HBSS). After adding 2 ml each of 78%, 69%, and 52% percoll gradient solution (17089102, GE Health care, Marlborough, USA 17089102) evenly and sequentially in tubes, bone marrow cells were centrifuged (1500 g, 30 min, 4 °C) in gradient solution without breaking. Neutrophil layers were collected at the bottom layer. The cells were subjected to two washes with phosphate-buffered saline PBS and then treated to achieve red blood cell lysis. Flow cytometry was utilized to evaluate the purity of the neutrophils obtained.

### Tumor models

Six-week-old female BALB/c mice were utilized. The 4T1 tumor model was established through orthotopic implantation, which involved injection of 1 × 10^6^ cells per mouse into the fourth left mammary fat pad. Subsequently, LDC7559 was administered daily over a four-week period. The 4T1 mouse breast carcinoma cell line, acquired from the American Type Culture Collection, was validated to be free from mycoplasma contamination. Tumor dimensions were evaluated at three-day intervals, with tumor volume determined using the formula: length × width^2^ × 0.5. All experimental procedures adhered to approved ethical standards. Upon reaching an average tumor volume of 0.114–0.117 cm³, the mice were randomly assigned to one of two treatment groups, each consisting of six mice: 1. Normal diet and 2. 20 mg/kg LDC7559. On day 30, the mice were euthanized, and the tumors were excised and frozen at −80 °C for further analysis. Primary tumors were injected through the caudal vein (3 × 10^5^ cells per mouse). After 7 days, Mito-TEMPO was administered continuously as a prophylactic treatment for a duration of one week using a subcutaneous pump.

To conduct the experimental metastasis assay, the E0771 mouse breast carcinoma cell line was acquired from the ATCC and confirmed to be free of mycoplasma contamination. A total of 2 × 10^5^ E0771 cells were intravenously injected into the tail vein of both wild-type (WT) and *Gsdmd*^*−/−*^ mice, which were kindly provided by Limin Lu from Fudan University. Following a two-week incubation period, the lungs were excised and fixed in 4% paraformaldehyde (PFA) for 24 h at a temperature of 4 °C. The fixed lung tissues were subsequently embedded in paraffin, allowed to air-dry at room temperature, and stained using hematoxylin and eosin (H&E) for histological examination.

### Single-cell RNA-seq data analysis

The single-cell sequencing data pertaining to triple-negative breast cancer were retrieved from the Gene Expression Omnibus dataset (GSE186910). Seurat was used for sequencing data analysis. The download sequencing data were processed using the Seurat (version 4.0.5) analysis package in R (V4.0). For each object, we filtered data based on the standard process, which is cells with fewer than 200 unique molecular identifiers (UMIs). Genes expression was then normalized using the default parameter of the Seurat package. FindVariableFeatures and ScaleData functions were used in the default parameter. The Seurat object was integrated using the FindIntegratedAnchors and IntegrateData functions in Seurat. Subsequently, Cell clustering was subsequently achieved through the application of the FindNeighbors function (dims = 1:8) and the FindClusters function (resolution = 0.8). The resulting clusters were visualized through Uniform Manifold Approximation and Projection (UMAP), which provided a comprehensive spatial depiction of the single cells. The aforementioned parameters were uniformly applied to all subsequent analyses of the single-cell datasets, ensuring consistency throughout the study.

### Data visualization

All plots were generated using the ggplot2 (v3.4.3) package in R 4.0.

### Flow cytometry analysis

Lung metastases were collected, minced, and digested in RPMI containing 10% FBS, 33.3 U/mL DNase I (Sigma D4513), and 0.5 mg/mL Collagenase IV (Sigma C5138) at 37 °C for 1 h. The cells were incubated with zombie (1:1000, Cat# 423102, Biolegend) at 4 °C for 25 min shielded from light to detect immune cells in the lungs for further analysis. For Fc receptor blocking, CD16/CD32 antibodies were applied in FACS buffer composed of 1× PBS supplemented with 0.5% FBS and 2 mM EDTA. The incubation was carried out at 4 °C for a duration of 30 min, protected from light. Following this, the cells were resuspended in FACS buffer containing the antibodies and incubated in the dark at 4 °C for 30 min. Data acquisition was performed utilizing a BD Canto II (BD Biosciences), and the resulting data were analyzed using FlowJo software (Tree Star, Inc.). The cells were then resuspended staining with Pacific Blue Rat anti-mouse Ly6G (Cat# 127612, Biolegend), PE-anti-mouse Ly6C (Cat# 128008, Biolegend), APC-anti-mouse CD11b (Cat# 101212, Biolegend), Bv421 anti-mouse CD4 (Cat# 100428, Biolegend) antibodies at 4 °C for 25 min shielded from light. As for hematopoietic cell detecting, bone marrow cells were harvested and subjected to red blood cell lysis. Cells were incubated with Zombie at 4 °C for 25 min shielded from light. The cells were then suspended in FACS buffer with lineage antibodies cocktail including Biotin-TER-119 (Cat# 116204, Biolegend), Biotin-CD11b (Cat# 101204, Biolegend), Biotin-CD3ε (Cat# 100304, Biolegend), Biotin-CD45R/B220 (Cat# 103204, Biolegend) and Biotin-IL-7Rα (Cat#135006, Biolegend) at 4 °C for 30 min in the dark. After washed once by PBS, cells were incubated with Percp-Cy5.5-Streptavidin (Cat# 405214, Biolegend), Pacific Blue-Sca-1 (clone D7, Biolegend), APC-c-Kit (Cat# 983302, Biolegend), APC-Cy7-CD16/32 (Cat# 101328, Biolegend) and AF647-CD34 (Cat# 128606, Biolegend) antibodies. Data were collected using a BD Canto II flow cytometer (BD Biosciences), followed by analysis with FlowJo software (Tree Star, Inc.).

### Quantification of cell-free DNA

Cell-free DNA was identified in the cultured medium, as previously detailed in reference [59]. For the detection of MPO-DNA and NE-DNA, 96-well plates were coated with anti-MPO antibody (Abcam, ab208670) and anti-neutrophil elastase antibody (Abcam, ab219585) in phosphate-buffered saline (PBS) and incubated at 4 °C overnight. After this incubation, a blocking buffer consisting of PBS with 5% bovine serum albumin (BSA) was introduced into the wells at room temperature for 1 h. The plates were then washed six times with wash buffer (PBS containing 0.05% Tween 20) before supernatants were added and allowed to incubate at room temperature for 2 h. After an additional round of washing with the wash buffer, the detection was carried out using PicoGreen from the Quant-iT PicoGreen dsDNA assay kit, in line with the manufacturer’s protocols (Invitrogen).

### Real-time quantitative PCR

Total RNA was extracted from the endplates using TRIzol (15596026, Invitrogen, Carlsbad, CA, USA), following the protocol provided by the manufacturer. Reverse transcription polymerase chain reaction (RT-PCR) was subsequently conducted with SYBR Green Master Mix (11201ES08, Yeasen) on a CFX Connect system (Bio-Rad, Hercules). Primer sequences used for targeting IL-1β, IL-18, and HMGB1 expression were: IL-1β: Forward: 5’-TGGACCTTCCAGGATGAGGACA-3’, Reverse: 5’-GTTCATCTCGGAGCCTGTAGTG-3’; IL-18: Forward: 5’-AAGCCAGAGCTGTGCAGATGAGTA-3’, Reverse: 5’-CTTGGTCACCGACGTCCTGT-3’; HMGB1: Forward: 5’-AAGGCTGGTCCATGCTCC-3’, Reverse: 5’-TGCTATCACTTCCTTTCTGTTGC-3’; GAPDH: Forward: 5’-TGGAAGGACTCATGACCACA-3’, Reverse: 5’-AGGGGTCTACATGGCAACTG-3’. MFN1: Forward: 5’- ATGGCAGAAACGGTATCTCCA-3’, Reverse: 5’-GCCCTCAGTAACAAACTCCAGT-3’. MFN2: Forward: 5’-AGAACTGGACCCGGTTACCA’, Reverse: 5’-CACTTCGCTGATACCCCTGA-3’, OPA1: Forward: 5’- TGGAAAATGGTTCGAGAGTCAG-3’, Reverse: 5’-CATTCCGTCTCTAGGTTAAAGCG-3’. Fis1: Forward: 5’-AGAGCACGCAATTTGAATATGCC-3’, Reverse: 5’-ATAGTCCCGCTGTTCCTCTTT-3’. TFAM: Forward: 5’-AACACCCAGATGCAAAACTTTCA-3’, Reverse: 5’-GACTTGGAGTTAGCTGCTCTTT-3’.

### Enzyme-linked immunosorbent assay (ELISA)

Mouse IL-18 (Invitrogen, BMS618-3), IL-1β (ELM-IL1b-1, RayBiotech) and HMGB1 (Elabscience, E-EL-M0676c) were detected using ELISA kits in the samples following the manufacturer’s protocols. Briefly, metastatic lung tissue was precisely sectioned into 0.2 g pieces and lysed with the specified lysis buffer. The lysates were then centrifuged at 1000 rpm for 15 min at a temperature of 4 °C. Following centrifugation, the samples were processed according to the manufacturers’ guidelines for the ELISA kits and subsequently analyzed using a microplate reader at a wavelength of 450 nm. The expression ratios were calculated using the formula provided in the instructions.

### Western blotting

Neutrophils were extracted from the wild-type (WT) lungs and 4T1 mice and subsequently lysed in a lysis buffer containing 50 mM Tris-HCl (pH 8.0), 2% sodium dodecyl sulfate (SDS), 10% glycerin, and protease inhibitors. The protein concentrations of the lysates were determined using a BCA Protein Assay Kit. Equal amounts of protein were analyzed through 10% sodium dodecyl sulfate-polyacrylamide gel electrophoresis (SDS-PAGE) and then transferred to polyvinylidene difluoride (PVDF) membranes (Merck Millipore Ltd, Burlington). The membranes underwent blocking with a solution of 5% bovine serum albumin in 1× TBST before being incubated with specific antibodies. Visualization of the proteins was accomplished using an enhanced chemiluminescence detection system.

### Transmission electron microscope

Neutrophils were extracted from the lung tissues of both control and 4T1 model mice and subsequently fixed in a solution of 2.5% glutaraldehyde dissolved in phosphate buffer at a temperature of 4 °C for a period of 2 h. Following fixation, the cells were rinsed twice with phosphate buffer and further fixed in 1% osmium tetroxide at 4 °C for an additional 2 h. The cells were then washed twice with distilled water. Dehydration was conducted stepwise using increasing concentrations of ethanol—30%, 50%, 70%, 80%, 95%, and 100%—with each concentration applied for 10 min. Ethanol was subsequently replaced with epoxypropane. The samples were then treated with a 1:1 mixture of Epon812 and epoxypropane for 2 h, followed by an overnight incubation with a 2:1 ratio of Epon812 to epoxypropane. Finally, pure epoxypropane was added and incubated at 37 °C for 6 h. The samples were then placed in a dryer for 48 h before being sectioned with an ultra-microtome (LEICA EM UC7). For electron staining, lead citrate was applied, and imaging was performed using a transmission electron microscope (H-7650, HITACHI).

### GSDMD oligomerization assay

GSDMD was assessed via Native gel electrophoresis, following the methodology previously described [20]. Neutrophils were isolated from the lung tissues of both control and 4T1 model mice. These cells were lysed using 250 μl of 5× SDS loading buffer, and the resulting lysates were evenly divided into two separate tubes. To create samples for non-reducing and reducing western blot analysis, TCEP or distilled deionized water (75 μl) was added to each tube. The samples were then heated at 65 °C for 10 min before being subjected to electrophoresis through a 12% NativePAGE Bis-Tris’s gel (Invitrogen) in NativePAGE running buffer (Invitrogen) at a temperature of 4 °C and a voltage of 150 V. After electrophoresis, the proteins were transferred to a polyvinylidene difluoride (PVDF) membrane using NativePAGE transfer buffer (Invitrogen) at a constant current of 0.25 A for a duration of 2 h to facilitate immunoblotting.

### NETs detection

Neutrophils were harvested from bone marrow and subsequently plated onto coverslips that had been pre-coated with poly-d-lysine (Corning, NY, USA). The induction of neutrophil extracellular traps (NETs) was conducted following the previously established protocol. The cells were fixed with 1% paraformaldehyde (PFA) in phosphate-buffered saline (PBS) and subsequently blocked using a solution of 1% bovine serum albumin (BSA) and 5% normal goat serum in PBS. The fixed cells were then incubated overnight at 4 °C with polyclonal rabbit neutrophil elastase in PBS. Following this incubation, the cells were washed and exposed to secondary antibodies (Life Technologies) diluted to 1:500 for a duration of 2 h. Sytox Green was prepared at a dilution of 1:500 and applied in the dark for 15 min to stain the cells. Imaging was carried out using an Olympus FV3000 microscope with a magnification of 20×.

### Immunofluorescence of metastatic lung tumors

Metastatic lung tumors were obtained and fixed in 4% paraformaldehyde (PFA) at a temperature of 4 °C for a duration of 12 h. Following fixation, the tumors were embedded in OCT compound, and sections were prepared for staining. The tumor sections were washed with PBS and subsequently permeabilized with 0.1% Triton X-100 for 20 min at room temperature. This was followed by blocking with a solution containing 3% goat serum and 3% FBS for 60 min at room temperature. The slides were incubated overnight at 4 °C with Alexa Fluor® 488 anti-mouse CD68 antibody (Cat#137011, BioLegend) and Alexa Fluor® 594 anti-mouse CD8a antibody (Cat# 100758, BioLegend). After washing the sections three times with PBS, they were stained with Hoechst 33342 (Cat#H3570, Thermo Fisher) for 15 min at room temperature. Imaging was conducted using an Olympus FV3000 microscope, and the resulting images were analyzed utilizing ImageJ software.

### Neutrophils immunofluorescent staining

Neutrophils were initially cultured at a density of 2× 10^6^ cells/mL on glass-bottom dishes that had been pre-coated with 0.001% poly-L-lysine, and this was carried out overnight. Following this incubation, the cells were stimulated and treated according to the specified experimental conditions. The neutrophils were subsequently fixed in 2% paraformaldehyde at 37 °C for a duration of 10 min. After fixation, the cells were washed with phosphate-buffered saline (PBS) and permeabilized using 0.1% Triton X-100 for 10 min at 4 °C. Following permeabilization, the cells underwent a blocking step with a solution containing 3% goat serum and 3% fetal bovine serum (FBS) for 1 h at 4 °C.

The staining protocol involved the application of primary antibodies, including GSDMD (ab219800, Abcam) and Did Plasma Membrane Stain (V22887, Thermo Fisher). After subjecting to three washes with PBS, cells were incubated at room temperature for two hours with the fluorescently labeled secondary antibody, specifically the Alexa Fluor® 555-conjugated Donkey anti-Rabbit IgG (H + L) (A0453, Beyotime Biotechnology). Following this incubation, the cells underwent three additional washes before being stained with DAPI for a duration of 20 minutes at room temperature. After additional three washes, the cells were incubated with Hoechst and Sytox Green (S7020, Thermo Fisher) for 15 min at room temperature. Imaging was conducted using an Olympus FV3000 microscope, and the images obtained were subsequently analyzed with ImageJ software.

### Immunofluorescence of tumor cells

4T1 cells were initially plated at a density of 2× 10^5^ cells/mL on glass-bottom dishes pre-coated with 0.001% poly-L-Lysine and then cultured with different neutrophil medium for two days. Subsequently, the tumor cells were fixed in 1% PFA at 37 °C for 10 min. Then cells underwent permeabilized in 0.1% Triton X-100 for 10 min at room temperature. The blocking step was carried out using a solution of 3% bovine serum albumin (BSA) and 3% goat serum diluted in phosphate-buffered saline for 40 min at room temperature. Following blocking, primary antibodies were utilized to incubate cells overnight at 4 °C with the appropriate.

Primary antibodies include E-cadherin antibody (ab231303, Abcam) and N-cadherin (ab98952, Abcam). After three washes with PBS, they were incubated at room temperature for 2 h with the fluorescently labeled secondary antibody, Alexa Fluor® 555-labeled anti-Rabbit IgG. Following three additional washes with PBS, the cells were stained with Hoechst for 15 min at room temperature. Imaging was conducted using an Olympus FV3000 microscope, and the obtained images were subsequently analyzed using ImageJ software.

### Statistical analysis

All methods were performed in accordance with the relevant guidelines and regulations. Statistical analyses were performed utilizing GraphPad Prism 8 (GraphPad Software). The results are reported as means ± standard error of the mean (SEM). An independent t-test was applied for comparisons between two groups, whereas one-way analysis of variance (ANOVA) followed by Tukey’s post-hoc test was employed for comparisons among more than two groups. Correlation analyses were conducted using Pearson’s correlation coefficient. A p-value of less than 0.05 was considered indicative of statistical significance.

### Ethic process

The research process was reviewed and approved by the ethics approval committee of the Laboratory Animal Administrative Committee of Shanghai Fourth People’s Hospital (approval ID: TJBH11924103). The animal experiment has passed the ethics certification organization of Tongji University, following the experimental animal care and experimental guidelines.

## Supplementary information

Supplemental material

## Data Availability

Nonprofit research will be approved for access for these data. All other raw data are available upon request from the corresponding author.

## References

[1] Carioli G, Malvezzi M, Rodriguez T, Bertuccio P, Negri E, La Vecchia C. Trends and predictions to 2020 in breast cancer mortality in Europe. Breast. 2017;36:89–95. 28988610 10.1016/j.breast.2017.06.003

[2] Sung H, Ferlay J, Siegel RL, Laversanne M, Soerjomataram I, Jemal A, et al. Global Cancer Statistics 2020: GLOBOCAN Estimates of Incidence and Mortality Worldwide for 36 Cancers in 185 Countries. CA Cancer J Clin. 2021;71:209–49. 33538338 10.3322/caac.21660

[3] Gray GK, Li CM, Rosenbluth JM, Selfors LM, Girnius N, Lin JR, et al. A human breast atlas integrating single-cell proteomics and transcriptomics. Dev Cell. 2022;57:1400–1420.e1407. 35617956 10.1016/j.devcel.2022.05.003 PMC9202341

[4] Jiang YZ, Ma D, Suo C, Shi J, Xue M, Hu X, et al. Genomic and Transcriptomic Landscape of Triple-Negative Breast Cancers: Subtypes and Treatment Strategies. Cancer Cell. 2019;35:428–440.e425. 30853353 10.1016/j.ccell.2019.02.001

[5] Nolan E, Lindeman GJ, Visvader JE. Deciphering breast cancer: from biology to the clinic. Cell. 2023;186:1708–28. 36931265 10.1016/j.cell.2023.01.040

[6] Kim C, Gao R, Sei E, Brandt R, Hartman J, Hatschek T, et al. Chemoresistance Evolution in Triple-Negative Breast Cancer Delineated by Single-Cell Sequencing. Cell. 2018;173:879–893.e813. 29681456 10.1016/j.cell.2018.03.041 PMC6132060

[7] Baldominos P, Barbera-Mourelle A, Barreiro O, Huang Y, Wight A, Cho JW, et al. Quiescent cancer cells resist T cell attack by forming an immunosuppressive niche. Cell. 2022;185:1694–1708.e1619. 35447074 10.1016/j.cell.2022.03.033 PMC11332067

[8] Costa A, Kieffer Y, Scholer-Dahirel A, Pelon F, Bourachot B, Cardon M, et al. Fibroblast Heterogeneity and Immunosuppressive Environment in Human Breast Cancer. Cancer Cell. 2018;33:463–479.e410. 29455927 10.1016/j.ccell.2018.01.011

[9] Wu SZ, Al-Eryani G, Roden DL, Junankar S, Harvey K, Andersson A, et al. A single-cell and spatially resolved atlas of human breast cancers. Nat Genet. 2021;53:1334–47. 34493872 10.1038/s41588-021-00911-1 PMC9044823

[10] Ethier JL, Desautels D, Templeton A, Shah PS, Amir E. Prognostic role of neutrophil-to-lymphocyte ratio in breast cancer: a systematic review and meta-analysis. Breast Cancer Res. 2017;19:2. 28057046 10.1186/s13058-016-0794-1 PMC5217326

[11] Yang L, Liu Q, Zhang X, Liu X, Zhou B, Chen J, et al. DNA of neutrophil extracellular traps promotes cancer metastasis via CCDC25. Nature. 2020;583:133–8. 32528174 10.1038/s41586-020-2394-6

[12] Xiao Y, Cong M, Li J, He D, Wu Q, Tian P, et al. Cathepsin C promotes breast cancer lung metastasis by modulating neutrophil infiltration and neutrophil extracellular trap formation. Cancer Cell. 2021;39:423–437.e427. 33450198 10.1016/j.ccell.2020.12.012

[13] Kayagaki N, Stowe IB, Lee BL, O’Rourke K, Anderson K, Warming S, et al. Caspase-11 cleaves gasdermin D for non-canonical inflammasome signalling. Nature. 2015;526:666–71. 26375259 10.1038/nature15541

[14] Shi J, Zhao Y, Wang K, Shi X, Wang Y, Huang H, et al. Cleavage of GSDMD by inflammatory caspases determines pyroptotic cell death. Nature. 2015;526:660–5. 26375003 10.1038/nature15514

[15] Ding J, Wang K, Liu W, She Y, Sun Q, Shi J, et al. Pore-forming activity and structural autoinhibition of the gasdermin family. Nature. 2016;535:111–6. 27281216 10.1038/nature18590

[16] Liu X, Zhang Z, Ruan J, Pan Y, Magupalli VG, Wu H, et al. Inflammasome-activated gasdermin D causes pyroptosis by forming membrane pores. Nature. 2016;535:153–8. 27383986 10.1038/nature18629 PMC5539988

[17] Miao N, Yin F, Xie H, Wang Y, Xu Y, Shen Y, et al. The cleavage of gasdermin D by caspase-11 promotes tubular epithelial cell pyroptosis and urinary IL-18 excretion in acute kidney injury. Kidney Int. 2019;96:1105–20. 31405732 10.1016/j.kint.2019.04.035

[18] Khanova E, Wu R, Wang W, Yan R, Chen Y, French SW, et al. Pyroptosis by caspase11/4-gasdermin-D pathway in alcoholic hepatitis in mice and patients. Hepatology. 2018;67:1737–53. 29108122 10.1002/hep.29645 PMC5906140

[19] Li S, Wu Y, Yang D, Wu C, Ma C, Liu X, et al. Gasdermin D in peripheral myeloid cells drives neuroinflammation in experimental autoimmune encephalomyelitis. J Exp Med. 2019;216:2562–81. 31467036 10.1084/jem.20190377 PMC6829591

[20] Miao N, Wang Z, Wang Q, Xie H, Yang N, Wang Y, et al. Oxidized mitochondrial DNA induces gasdermin D oligomerization in systemic lupus erythematosus. Nat Commun. 2023;14:872. 36797275 10.1038/s41467-023-36522-z PMC9935630

[21] Kloosterman DJ, Erbani J, Boon M, Farber M, Handgraaf SM, Ando-Kuri M, et al. Macrophage-mediated myelin recycling fuels brain cancer malignancy. Cell. 2024:187:5336–5356.e30. 10.1016/j.cell.2024.07.030 PMC11429458 39137777

[22] He XY, Gao Y, Ng D, Michalopoulou E, George S, Adrover JM, et al. Chronic stress increases metastasis via neutrophil-mediated changes to the microenvironment. Cancer Cell. 2024;42:474–486.e412. 38402610 10.1016/j.ccell.2024.01.013 PMC11300849

[23] Evavold CL, Hafner-Bratkovic I, Devant P, D’Andrea JM, Ngwa EM, Borsic E, et al. Control of gasdermin D oligomerization and pyroptosis by the Ragulator-Rag-mTORC1 pathway. Cell. 2021;184:4495–511.e4419. 34289345 10.1016/j.cell.2021.06.028 PMC8380731

[24] Sollberger G, Choidas A, Burn GL, Habenberger P, Di Lucrezia R, Kordes S, et al. Gasdermin D plays a vital role in the generation of neutrophil extracellular traps. Sci. Immunol. 2018;3:eaar6689. 10.1126/sciimmunol.aar6689 30143555

[25] Le Naour A, Koffi Y, Diab M, Le Guennec D, Rouge S, Aldekwer S, et al. EO771, the first luminal B mammary cancer cell line from C57BL/6 mice. Cancer Cell Int. 2020;20:328. 32699527 10.1186/s12935-020-01418-1 PMC7372867

[26] Arner EN, Rathmell JC. Metabolic programming and immune suppression in the tumor microenvironment. Cancer Cell. 2023;41:421–33. 36801000 10.1016/j.ccell.2023.01.009 PMC10023409

[27] Segal BH, Giridharan T, Suzuki S, Khan ANH, Zsiros E, Emmons TR, et al. Neutrophil interactions with T cells, platelets, endothelial cells, and of course tumor cells. Immunol Rev. 2022;314:13–35. 10.1111/imr.13178 PMC10174640 36527200

[28] Amara N, Cooper MP, Voronkova MA, Webb BA, Lynch EM, Kollman JM, et al. Selective activation of PFKL suppresses the phagocytic oxidative burst. Cell. 2021;184:4480–4494 e4415. 34320407 10.1016/j.cell.2021.07.004 PMC8802628

[29] Yu E, Zhang E, Lv X, Yan L, Lin Z, Siaw-Debrah F, et al. LDC7559 Exerts Neuroprotective Effects by Inhibiting GSDMD-Dependent Pyroptosis of Microglia in Mice with Traumatic Brain Injury. J Neurotrauma. 2023;40:742–57. 10.1089/neu.2021.0318 35920115

[30] Casbon AJ, Reynaud D, Park C, Khuc E, Gan DD, Schepers K, et al. Invasive breast cancer reprograms early myeloid differentiation in the bone marrow to generate immunosuppressive neutrophils. Proc Natl Acad Sci USA. 2015;112:E566–575. 25624500 10.1073/pnas.1424927112 PMC4330753

[31] Ubellacker JM, Baryawno N, Severe N, DeCristo MJ, Sceneay J, Hutchinson JN, et al. Modulating Bone Marrow Hematopoietic Lineage Potential to Prevent Bone Metastasis in Breast Cancer. Cancer Res. 2018;78:5300–14. 30065048 10.1158/0008-5472.CAN-18-0548 PMC6309204

[32] Pilla DM, Hagar JA, Haldar AK, Mason AK, Degrandi D, Pfeffer K, et al. Guanylate binding proteins promote caspase-11-dependent pyroptosis in response to cytoplasmic LPS. Proc Natl Acad Sci USA. 2014;111:6046–51. 24715728 10.1073/pnas.1321700111 PMC4000848

[33] Xie F, Zhou X, Su P, Li H, Tu Y, Du J, et al. Breast cancer cell-derived extracellular vesicles promote CD8(+) T cell exhaustion via TGF-beta type II receptor signaling. Nat Commun. 2022;13:4461. 35915084 10.1038/s41467-022-31250-2 PMC9343611

[34] Grasset EM, Dunworth M, Sharma G, Loth M, Tandurella J, Cimino-Mathews A, et al. Triple-negative breast cancer metastasis involves complex epithelial-mesenchymal transition dynamics and requires vimentin. Sci Transl Med. 2022;14:eabn7571. 35921474 10.1126/scitranslmed.abn7571 PMC9801390

[35] Papayannopoulos V. Neutrophil extracellular traps in immunity and disease. Nat Rev Immunol. 2018;18:134–47. 28990587 10.1038/nri.2017.105

[36] Park J, Wysocki RW, Amoozgar Z, Maiorino L, Fein MR, Jorns J, et al. Cancer cells induce metastasis-supporting neutrophil extracellular DNA traps. Sci Transl Med. 2016;8:361ra138. 27798263 10.1126/scitranslmed.aag1711 PMC5550900

[37] Teijeira A, Garasa S, Gato M, Alfaro C, Migueliz I, Cirella A, et al. CXCR1 and CXCR2 chemokine receptor agonists produced by tumors induce neutrophil extracellular traps that interfere with immune cytotoxicity. Immunity. 2020;52:856–71 e858. 32289253 10.1016/j.immuni.2020.03.001

[38] Xia X, Zhang Z, Zhu C, Ni B, Wang S, Yang S, et al. Neutrophil extracellular traps promote metastasis in gastric cancer patients with postoperative abdominal infectious complications. Nat Commun. 2022;13:1017. 35197446 10.1038/s41467-022-28492-5 PMC8866499

[39] Sorensen OE, Borregaard N. Neutrophil extracellular traps - the dark side of neutrophils. J Clin Invest. 2016;126:1612–20. 27135878 10.1172/JCI84538 PMC4855925

[40] Albrengues J, Shields MA, Ng D, Park CG, Ambrico A, Poindexter ME, et al. Neutrophil extracellular traps produced during inflammation awaken dormant cancer cells in mice. Science. 2018; 361:6409. 10.1126/science.aao4227 PMC6777850 30262472

[41] Song R, Han X, Jie H, Zhang X, Li S, Sun E. Effects and mechanisms of Celastrol on the formation of neutrophil extracellular traps (NETs). Ann Transl Med. 2023;11:16. 36760253 10.21037/atm-22-5720 PMC9906213

[42] Zhang Y, Yang Y, Hu X, Wang Z, Li L, Chen P. PADs in cancer: current and future. Biochim Biophys Acta Rev Cancer. 2021;1875:188492. 33321174 10.1016/j.bbcan.2020.188492

[43] Gong Z, Li Q, Shi J, Wei J, Li P, Chang CH, et al. Lung fibroblasts facilitate pre-metastatic niche formation by remodeling the local immune microenvironment. Immunity. 2022;55:1483–1500 e1489. 35908547 10.1016/j.immuni.2022.07.001 PMC9830653

[44] Muscarella AM, Aguirre S, Hao X, Waldvogel SM, Zhang XH. Exploiting bone niches: progression of disseminated tumor cells to metastasis. J Clin Invest. 2021;131:e143764. 10.1172/JCI143764 PMC7954594 33720051

[45] Weng YS, Tseng HY, Chen YA, Shen PC, Al Haq AT, Chen LM, et al. MCT-1/miR-34a/IL-6/IL-6R signaling axis promotes EMT progression, cancer stemness and M2 macrophage polarization in triple-negative breast cancer. Mol Cancer. 2019;18:42. 30885232 10.1186/s12943-019-0988-0 PMC6421700

[46] DeNardo DG, Ruffell B. Macrophages as regulators of tumour immunity and immunotherapy. Nat Rev Immunol. 2019;19:369–82. 30718830 10.1038/s41577-019-0127-6 PMC7339861

[47] Zhang Y, Guo L, Dai Q, Shang B, Xiao T, Di X, et al. A signature for pan-cancer prognosis based on neutrophil extracellular traps. J Immunother Cancer. 2022;10:e004210. 10.1136/jitc-2021-004210 PMC9189842 35688556

[48] Ye F, Dewanjee S, Li Y, Jha NK, Chen ZS, Kumar A, et al. Advancements in clinical aspects of targeted therapy and immunotherapy in breast cancer. Mol Cancer. 2023;22:105. 37415164 10.1186/s12943-023-01805-y PMC10324146

[49] Arora S, Narayan P, Osgood CL, Wedam S, Prowell TM, Gao JJ, et al. U.S. FDA drug approvals for breast cancer: a decade in review. Clin Cancer Res. 2022;28:1072–86. 34711632 10.1158/1078-0432.CCR-21-2600 PMC8923912

[50] Safarulla S, Madan A, Xing F. Chandrasekaran A: CXCR2 mediates distinct neutrophil behavior in brain metastatic breast tumor. Cancers. 2022;14:515. 10.3390/cancers14030515 PMC8833752 35158784

[51] Kanneganti A, Malireddi RKS, Saavedra PHV, Vande Walle L, Van Gorp H, Kambara H, et al. GSDMD is critical for autoinflammatory pathology in a mouse model of Familial Mediterranean Fever. J Exp Med. 2018;215:1519–29. 29793924 10.1084/jem.20172060 PMC5987922

[52] Heilig R, Dick MS, Sborgi L, Meunier E, Hiller S, Broz P. The Gasdermin-D pore acts as a conduit for IL-1beta secretion in mice. Eur J Immunol. 2018;48:584–92. 29274245 10.1002/eji.201747404

[53] Shi J, Gao W, Shao F. Pyroptosis: gasdermin-mediated programmed necrotic cell death. Trends Biochemical Sci. 2017;42:245–54. 10.1016/j.tibs.2016.10.004 27932073

[54] Xu J, Nunez G. The NLRP3 inflammasome: activation and regulation. Trends Biochemical Sci. 2023;48:331–44. 10.1016/j.tibs.2022.10.002 PMC10023278 36336552

[55] Zhao Z, Fang L, Xiao P, Sun X, Zhou L, Liu X, et al. Walking dead tumor cells for targeted drug delivery against lung metastasis of triple-negative breast cancer. Adv Mater. 2022;34:e2205462. 35759925 10.1002/adma.202205462

[56] Duan X, Zhang Q, Jiang Y, Wu X, Yue X, Geng Y, et al. Semiconducting polymer nanoparticles with intramolecular motion-induced photothermy for tumor phototheranostics and tooth root canal therapy. Adv Mater. 2022;34:e2200179. 35239994 10.1002/adma.202200179

[57] Maris P, Blomme A, Palacios AP, Costanza B, Bellahcene A, Bianchi E, et al. Asporin is a fibroblast-derived TGF-beta1 inhibitor and a tumor suppressor associated with good prognosis in breast cancer. PLoS Med. 2015;12:e1001871. 26327350 10.1371/journal.pmed.1001871 PMC4556693

[58] Shen Q, Cohen B, Zheng W, Rahbar R, Martin B, Murakami K, et al. Notch shapes the innate immunophenotype in breast cancer. Cancer Discov. 2017;7:1320–35. 28790030 10.1158/2159-8290.CD-17-0037

[59] Hu S, Liu X, Gao Y, Zhou R, Wei M, Dong J, et al. Hepatitis B virus inhibits neutrophil extracellular trap release by modulating reactive oxygen species production and autophagy. J Immunol. 2019;202:805–15. 30567731 10.4049/jimmunol.1800871

