# Supplementary material for: Mitochondrial reactive oxygen species promote cancer metastasis and tumor microenvironment immunosuppression through gasdermin D
Source: Cell Death Discov. 2025 May 6;11:219. doi: 10.1038/s41420-025-02516-7 (PMC12053750; doi:10.1038/s41420-025-02516-7)
Supplement: Supplementary file 1 — Supplemental material [file 41420_2025_2516_MOESM1_ESM.docx]

**Mitochondrial reactive oxygen species promote cancer metastasis and tumor microenvironment immunosuppression through gasdermin D**

Naijun Miao^1,2#^, Zhengchun Kang^3#^, Zhuning Wang^2#^, Wenyan Yu^1#^, Ting Liu^4^, Ling-zhijie Kong^1^, Ying Zheng^1^, Changli Ding^1^, Zhiyong Zhang^1^, Chen Zhong^5^*, Qingliang Fang^6^*, Kaichun Li^1^*

^1^Department of Oncology, Shanghai Fourth People's Hospital, Tongji University School of Medicine, Shanghai, China, 200434

^2^Center for Immune-related Diseases at Shanghai Institute of Immunology, Ruijin Hospital, Shanghai Jiao Tong University School of Medicine, Shanghai, China, 200025

^3^Department of Colorectal Surgery, Changhai Hospital, Naval Medical University, Shanghai, China, 200433

^4^Department of Anesthesiology, Ruijin Hospital, Shanghai Jiao Tong University School of Medicine, Shanghai, China, 200025

^5^Department of Medical Oncology, The 960th Hospital of the PLA Joint Logistice Support Force, Jinan, Shandong, China, 250031

^6^Department of Radiation Oncology, LongHua Hospital Shanghai University of Traditional Chinese Medicine, Shanghai, China, 200032

^#^These authors contributed equally to this work

^*^Corresponding Author: Kaichun Li, M.D. E-mail: likaichun@tongji.edu.cn. No.1279, Sanmen Road, Hongkou District, Shanghai, China, 200434.

Qingliang Fang. E-mail: [fangqingliang@shutcm.edu.cn](mailto:fangqingliang@shutcm.edu.cn).

Chen Zhong, E-mail: zhongchen0504@sina.com.

**Supplemental Figures**

**
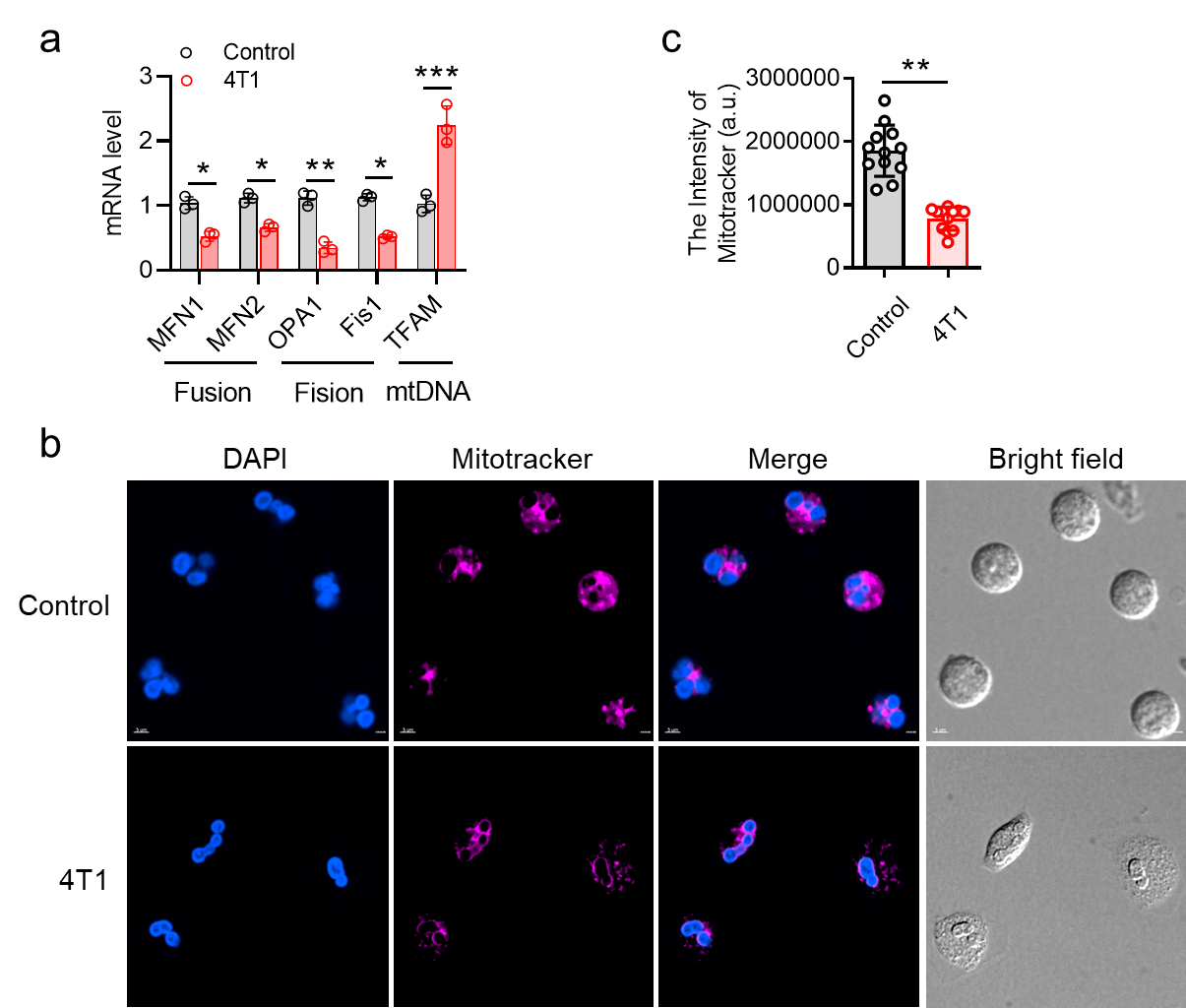
**

**Supplementary Figure 1. The Mitochondrial quality control in lung neutrophils from breast cancer metastatic mouse model.** (a) The mRNA level of MFN1, MFN2, OPA1, Fis1 and TFAM in lung neutrophils form control and 4T1 mice. (b) Immunofluorescent of Mitotracker staining in lung neutrophils form control and 4T1 mice. Scal bar, 5 μm. (c) Quantitative analysis of intensity of Mitotracker in (b). Data are presented as means+SEM. Significance was examined with Student’s *t*-test (a, c). ^*^*P*<0.05, ^**^*P*<0.01, ^***^*P*<0.001.


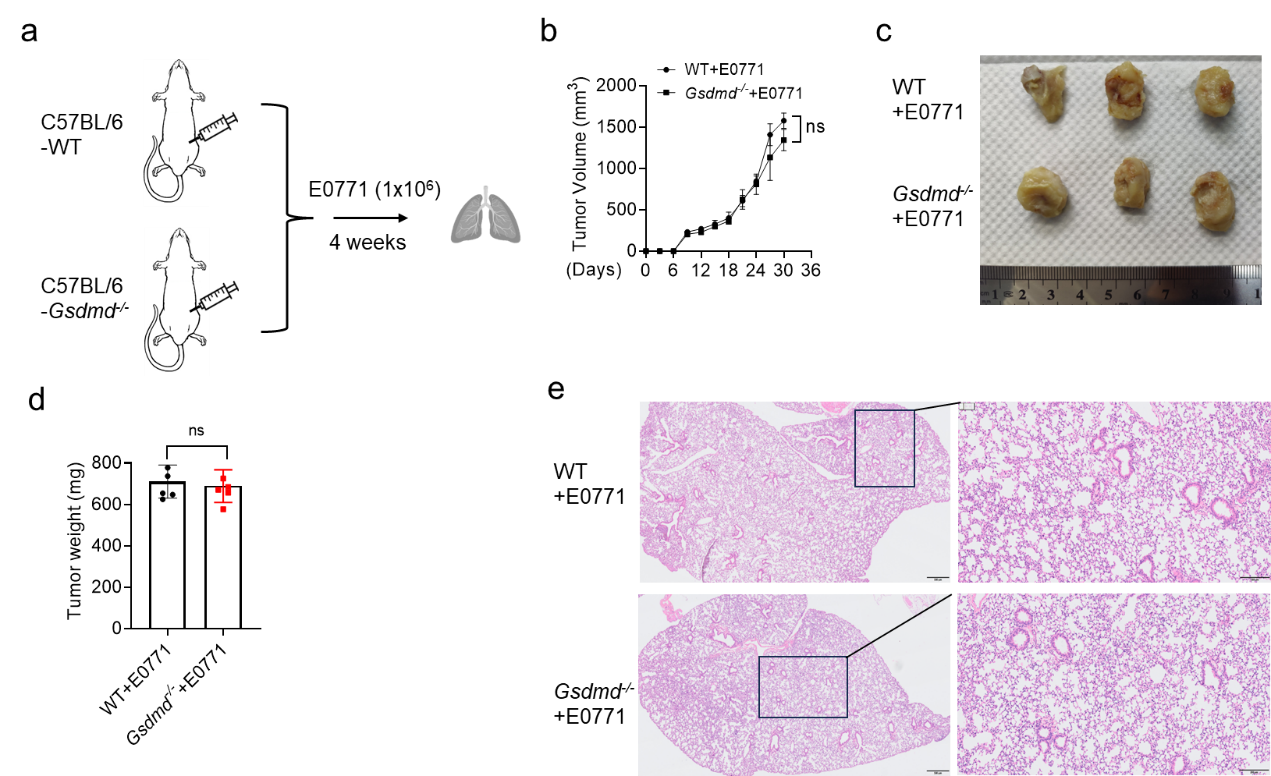


**Supplementary Figure 2. The tumor progression and metastasis were not blunted in *Gsdmd^-/-^* mice after E0771 orthotopic injection.** (a) The flow chat of the experiment. (b) Photograph of tumor from WT and *Gsdmd^-/-^* mice after E0771 inoculation. (c) Quantitative analysis of tumor weight. (d) Quantitative analysis of tumor volume from 6 to 36 days after E0771 inoculation. (e) H&E staining of lung from WT and *Gsdmd^-/-^* mice after E0771 inoculation. Scale bar, 500 μm, 200 μm (enlarged). Data are presented as means+SEM. Significance was examined with Student’s *t*-test (c, d). ns, not significant.


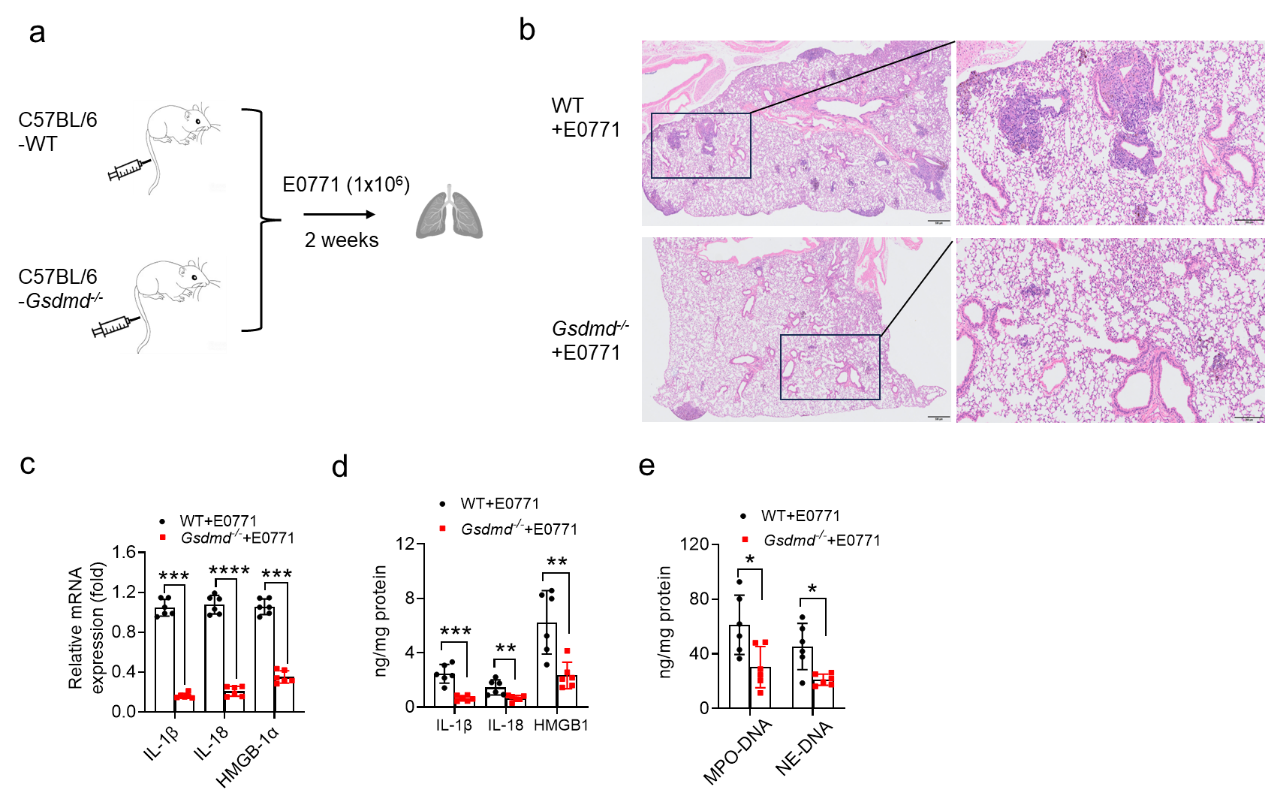


**Supplementary Figure 3. The lung metastasis was significantly reduced in *Gsdmd^-/-^* mice after E0771 intravenous injection.** (a) The flow chat of the experiment. (b) H&E staining of lung from WT and *Gsdmd^-/-^* mice after E0771 intravenous injection. Scale bar, 500 μm, 200 μm (enlarged). (c) Quantitative analysis of the metastatic nodules per lobe. (d) mRNA level of IL-1β, IL-18 and HMGB-1 in lung. (e) ELISA analysis of IL-1β, IL-18 and HMGB-1 in lung. (f) ELISA analysis of MPO-DNA and NE-DNA in serum. Data are presented as mean+SEM. Significance was examined by one-way AVOVA (c) or two-tailed unpaired Student’s t-test (d, e, f). ^*^*P*<0.05, ^**^*P*<0.01, ^***^*P*<0.001. ^****^*P*<0.0001.


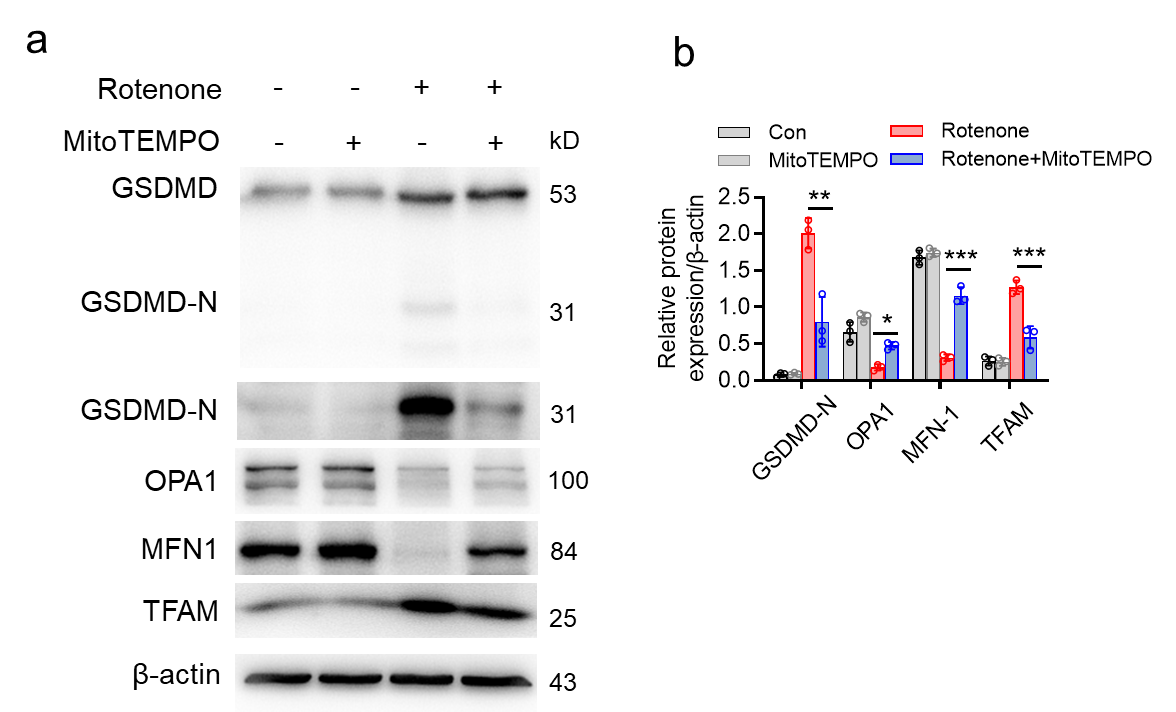


**Supplementary Figure 4. mROS significantly promotes GSDMD activation.** (a) Western blot anlaysis of GSDMD, GSDMD-N, OPA1, MFN1 and TFAM in mouse bone marrow neutrophils after Rotenone or Rotenone with MitoTEMPO treatment. (b) Quantitative analysis of GSDMD-N, OPA1, MFN1 and TFAM in (a). Data are presented as mean+SEM. Significance was examined by two-way AVOVA (b). ^*^*P*<0.05, ^**^*P*<0.01, ^***^*P*<0.001.
